# Sustained immune activation and impaired epithelial barrier integrity in the ectocervix of women with chronic HIV infection

**DOI:** 10.1371/journal.ppat.1012709

**Published:** 2024-11-19

**Authors:** Mathias Franzén Boger, Tyra Hasselrot, Vilde Kaldhusdal, Gisele H. B. Miranda, Paulo Czarnewski, Gabriella Edfeldt, Frideborg Bradley, Genta Rexaj, Julie Lajoie, Kenneth Omollo, Joshua Kimani, Keith R. Fowke, Kristina Broliden, Annelie Tjernlund

**Affiliations:** 1 Division of Infectious Diseases, Department of Medicine Solna, Karolinska Institutet, Karolinska University Hospital, Center for Molecular Medicine, Stockholm, Sweden; 2 Division of Computational Science and Technology, KTH Royal Institute of Technology, Stockholm, Sweden; 3 BioImage Informatics Facility, Science for Life Laboratory, Solna, Sweden; 4 Science for Life Laboratory, Department of Biochemistry and Biophysics and National Bioinformatics Infrastructure Sweden, Stockholm University, Stockholm, Sweden; 5 Department of Medical Microbiology and Infectious Diseases, University of Manitoba, Winnipeg, Canada; 6 Department of Medical Microbiology and Immunology, University of Nairobi, Nairobi, Kenya; 7 Partners for Health and Development in Africa, Nairobi, Kenya; 8 Department of Community Health Sciences, University of Manitoba, Winnipeg, Canada; Burnet Institute, AUSTRALIA

## Abstract

Chronic systemic immune activation significantly influences human immunodeficiency virus (HIV) disease progression. Despite evidence of a pro-inflammatory environment in the genital tract of HIV-infected women, comprehensive investigations into cervical tissue from this region remain limited. Similarly, the consequences of chronic HIV infection on the integrity of the female genital epithelium are poorly understood, despite its importance in HIV transmission and replication. Ectocervical biopsies were obtained from HIV-seropositive (n = 14) and HIV-seronegative (n = 47) female Kenyan sex workers. RNA sequencing and bioimage analysis of epithelial junction proteins (E-cadherin, desmoglein-1, claudin-1, and zonula occludens-1) were conducted, along with CD4 staining. RNA sequencing revealed upregulation of immunoregulatory genes in HIV-seropositive women, primarily associated with heightened T cell activity and interferon signaling, which further correlated with plasma viral load. Transcription factor analysis confirmed the upregulation of pro-inflammatory transcription factors, such as *RELA*, *NFKB1*, and *IKZF3*, which facilitates HIV persistence in T cells. Conversely, genes and pathways associated with epithelial barrier function and structure were downregulated in the context of HIV. Digital bioimage analysis corroborated these findings, revealing significant disruption of various epithelial junction proteins in ectocervical tissues of the HIV-seropositive women. Thus, chronic HIV infection associated with ectocervical inflammation, characterized by induced T cell responses and interferon signaling, coupled with epithelial disruption. These alterations may influence HIV transmission and heighten susceptibility to other sexually transmitted infections. These findings prompt exploration of therapeutic interventions to address HIV-related complications and mitigate the risk of sexually transmitted infection transmission.

## Author summary

Chronic immune activation is a key feature of human immunodeficiency virus (HIV) infection and is linked to various health issues, including sexually transmitted infections. The consequence of immune activation is becoming evident as age-related morbidities are appearing in younger people living with HIV. The female genital tract is a primary location for initial HIV infection and replication following sexual transmission. While we know that chronic HIV infection causes inflammation in the tissue, more detailed studies, especially focusing on mucosal epithelial tissue, are still needed. To understand how chronic HIV infection affects the female genital tract, we examined samples from HIV-positive and HIV-negative Kenyan female sex workers. We found that chronic HIV infection is associated with a sustained cervical inflammatory environment. We also noticed a decrease in genes responsible for maintaining the protective epithelial barrier in the tissue. Our results suggest a connection between HIV-induced immune responses in mucosal areas and the health of the tissue, which might explain why HIV infection may increase the risk of other sexually transmitted infections. This highlights the importance of enhancing mucosal health in people living with chronic HIV to reduce associated complications.

## Introduction

Chronic systemic immune activation is a hallmark of human immunodeficiency virus (HIV) infection, serving as a primary driver of CD4^+^ T cell depletion and subsequent disease pathogenesis [1–4]. The consequence of systemic immune activation is becoming more evident as disorders that typically affect an aged population are appearing in younger individuals living with HIV [5,6]. The precise cause of this immune activation remains incompletely understood, though it likely stems from a combination of factors [3,7–9]. However, microbial translocation within the gastrointestinal (GI) tract, attributed to the rapid loss of CD4^+^ T cells and significant epithelial disruption, has emerged as a major contributor to HIV-mediated immune activation [10].

Several studies have documented elevated pro-inflammatory cytokines in cervicovaginal lavage and dysregulated immune cell populations in cells from cytobrush samples in HIV infected women [11–14]. The extent of CD4^+^ T cell depletion within the cervix remains incompletely elucidated. While studies on cytobrush-collected cervical mucosal mononuclear cells indicate a significant depletion of cervical CD4^+^ T cells, mirroring the depletion observed in the blood [15], depletion of CD4^+^ cells was not observed within the cervical tissues of HIV-infected women [16].

The ectocervical mucosa features a multilayered, stratified squamous epithelium interconnected by various intercellular junctions. These proteins collectively form a net-like structure, preserving the epithelium’s physiological characteristics and serving as a crucial physical barrier against the external environment [17]. Despite this protective function, *in vitro* studies have demonstrated the disruption of intercellular epithelial junctions following exposure to HIV, potentially facilitating viral transmigration and subsequent infection [18–20].

Nevertheless, the majority of these studies have relied on primary cell cultures or tissue explants, employing laboratory HIV strains or viral glycoproteins. Furthermore, the focus has primarily centered on epithelial disruption during acute HIV exposure, leaving the effects of chronic infection largely unexplored, particularly regarding its impact on the protective mucosal barrier lining the female genital tract (FGT). Similarly, as human tissue is hard to access, with an intricate processing procedure, much of the available data concerning the FGT mucosal immune response to chronic HIV has been derived from cervicovaginal lavage or genital mucosal mononuclear cells collected by cytobrush [12,14,21,22].

These methods provide only a limited perspective on the mucosa’s full complexity, offering a narrow view of the cellular and molecular interactions within the broad mucosal environment. Therefore, a more comprehensive characterization of the molecular alterations during chronic HIV infection in the FGT is imperative to advance our understanding of related complications, including sexually transmitted infections (STIs) and cervical cancer. These are pressing concerns given the disproportionate impact of HIV infection on women relative to men, attributed to both socioeconomic and biological factors, including a larger genital mucosal surface area, potentially contributing to the higher rate of HIV acquisition in women [23].

In this study, the aim was to deepen our understanding of the impact of chronic HIV infection on cervical tissue immunity and to uncover potential mechanistic clues explaining the elevated prevalence of other sexually transmitted infections (STIs) in women with chronic HIV infection [24–26]. Through comprehensive investigation of the ectocervical transcriptional landscape and quantitative bioimage analysis, we sought to achieve this goal by exploring the structure and function of a unique set of ectocervical tissue biopsies obtained from women with chronic HIV infection. The results presented herein demonstrate that our study successfully met its aims, elucidating the intricate molecular landscape of chronic HIV infection in the FGT. These findings provide valuable insights into potential therapeutic interventions to address HIV-related complications and mitigate the risk of STIs.

## Results

### Cohort description

This study comprised both HIV-seropositive and -seronegative female sex workers (HIV^+^FSW and HIV^–^FSW, respectively). Participants were selected from the Pumwani Sex Worker Cohort in Nairobi, Kenya as part of a larger longitudinal study [27]. We aimed for a 1:3 ratio of HIV^+^FSWs to HIV^–^FSWs, matching for age and duration of sex work. In total, 14 HIV^+^FSWs and 47 HIV^–^FSWs were included, with median ages of 38 and 36 years, respectively. The median duration of sex work was 8 years for both groups and the median time since HIV diagnosis was 47 months. All study participants were antiretroviral naïve and not receiving pre-exposure prophylaxis in line with national guidelines at time of the study. Sampling was aimed at the follicular phase of the menstrual cycle based on self-reported time since last menses which was further validated by plasma estradiol and progesterone levels. Among the HIV^+^FSWs, two were using the hormonal contraceptive depot medroxyprogesterone acetate (DMPA), while none of the HIV^-^FSWs did (P = 0.05). No differences could be observed in the sexual behavior of the study groups based on self-reported number of sexual clients during the week prior to the 2 weeks’ sexual abstinence period, or the frequency of participants with a regular partner. Furthermore, study participants were required to test negative for *Chlamydia trachomatis*, *Neisseria gonorrhoeae*, *Trichomonas vaginalis* and *Treponema pallidum* as per study enrollment criteria. At time of the present sample collection, all participants were retested for these STIs and confirmed negative. The incidence of bacterial vaginosis was similar across the groups and no differences were observed for the cervicovaginal microbiome composition based on 16S rRNA sequencing (**Table 1**) [28].

**Table 1:** Characteristics of cohort at time of sample collection.

Variables	HIV^+^FSWs n = 14 Median (range or %)	HIV^-^FSWs n = 47 Median (range or %)	P-value
**Age** (years)	38 [29–48]	36 [23–50]	0.19^1^
**Time in sex work**^**a**^ (months) • Not available	96 (24–180) 1 (7%)	96 (12–372) 0	0.53^1^
**Time since diagnosis**^**b**^ (months)	47 [9–78]	NA	NA
**Clients in the week prior to sexual abstinence**^**c**^ (number)	7 (0–30)	4 (0–50)	0.25^1^
**Regular partner**^**d**^ Yes	8 (57%)	23 (49%)	0.76^2^
**DMPA use**^**e**^ Yes	2 (14%)	0	0.05^2^
**Days since menses** ^**f**^	10 [4–22]	9 [3–44]	0.581
**Estradiol levels** (Plasma; pg/ml) Not available	85 (22–215) 1 (7%)	82 (22–405) 0	0.28^1^
**Progesterone levels** (Plasma; ng/ml) Not available	0.05 (0.05–8) 1 (7%)	0.05 (0.05–19) 0	0.90^1^
**Bacterial Vaginosis** BV; Nugent score • BV; 7–10 • Intermediate; 4–6 • Normal; 0–3	4 (29%) 3 (21%) 7 (50%)	12 (26%) 15 (32%) 20 (43%)	0.8^2^ 0.99^2^ 0.52^2^ 0.76^2^
**Cervicovaginal microbiome**^**g**^ • L1 • L2 • L3 • L4 • L5 Not available	1 (7%) 3 (21%) 2 (14%) 5 (36%) 2 (14%) 1 (7%)	5 (11%) 12 (26%) 11 (23%) 17 (36%) 1 (2%) 1 (2%)	0.99^2^ 0.99^2^ 0.71^2^ 0.99^2^ 0.13^2^ 0.41^2^
**STI screening**^**h**^ No	14 (100%)	47 (100%)	NA
**CD4 cell count** (cells/ml)	592 (347–749)	908 (326–1,439)	<0.001^1^
**HIV RNA** (copies/ml)	1,406 (0–276,000)	NA	NA

^a^Self-reported time in sex work.

^b^If already HIV seropositive at time of inclusion in the Pumwani sex worker cohort: Time since inclusion in the cohort.

^c^All participants had agreed to, and were followed up for, two weeks of abstinence from sex work prior to and after the sampling time point. Number of clients in the week prior to sexual abstinence was self-reported.

^d^Regular partner indicates having a regular partner in addition to other sex work clients (self-reported).

^e^DMPA: Regular use (since more than 6 months) of depot medroxyprogesterone acetate. The non-DMPA users did not use any type of hormonal contraceptives.

^f^Days since the first day of the most recent menstrual period. Participants using DMPA are not included.

^*g*^Cervicovaginal luminal microbiome (16S rRNA), as previously defined [28] as well as obtained from the Gene Expression Omnibus GSE217237. L1; > 80% abundance of *L*. *crispatus* and/or *L*. *Jensenii*. L2; > 80% *L*. spp. L3; > 10% *Gardnerella vaginalis* and/or < 5% *Prevotella*. L4; > 5% *Prevotella*. L5; Samples that did not fit within L1-L4.

^h^STI screening at time of sample collection: Diagnosis of any of the sexually transmitted infections *Chlamydia Trachomatis*, *Neisseria Gonorrhoeae*, *Treponema Pallidum* and *Trichomonas Vaginalis* was an exclusion criterium at study enrolment. These diagnostic tests were repeated at time of sample collection for the present sub-study.

FSW: female sex worker. NA: Not applicable. DMPA: Depot medroxyprogesterone acetate. BV: Bacterial vaginosis.

P-values: ^1^Mann Whitney U test; ^2^Fisher’s exact test.

### HIV infection was associated with differential gene expression of immunoregulatory and epithelial barrier-related genes

RNA sequencing (RNA-seq) data from ectocervical biopsies containing both epithelial and submucosal tissue of HIV^+^FSWs (n = 14) compared to HIV^–^FSWs (n = 47) were obtained from the Gene Expression Omnibus (GEO; GSE217237) to comprehensively characterize the molecular changes occurring within the FGT during chronic HIV infection in an unbiased manner. Out of 15,435 genes detected in the RNA-seq dataset, 320 exhibited significant differential expression with a false discovery rate (FDR)-adjusted P-value < 0.05 in HIV^+^FSWs compared to HIV^–^FSWs. Among these 320 differentially expressed genes (DEGs), 183 were upregulated in HIV^+^FSWs compared to HIV^–^FSWs (log_2_FC: +4.2 to +0.2), while 137 were downregulated (log_2_FC: -4.5 to -0.3).

**Fig 1:**
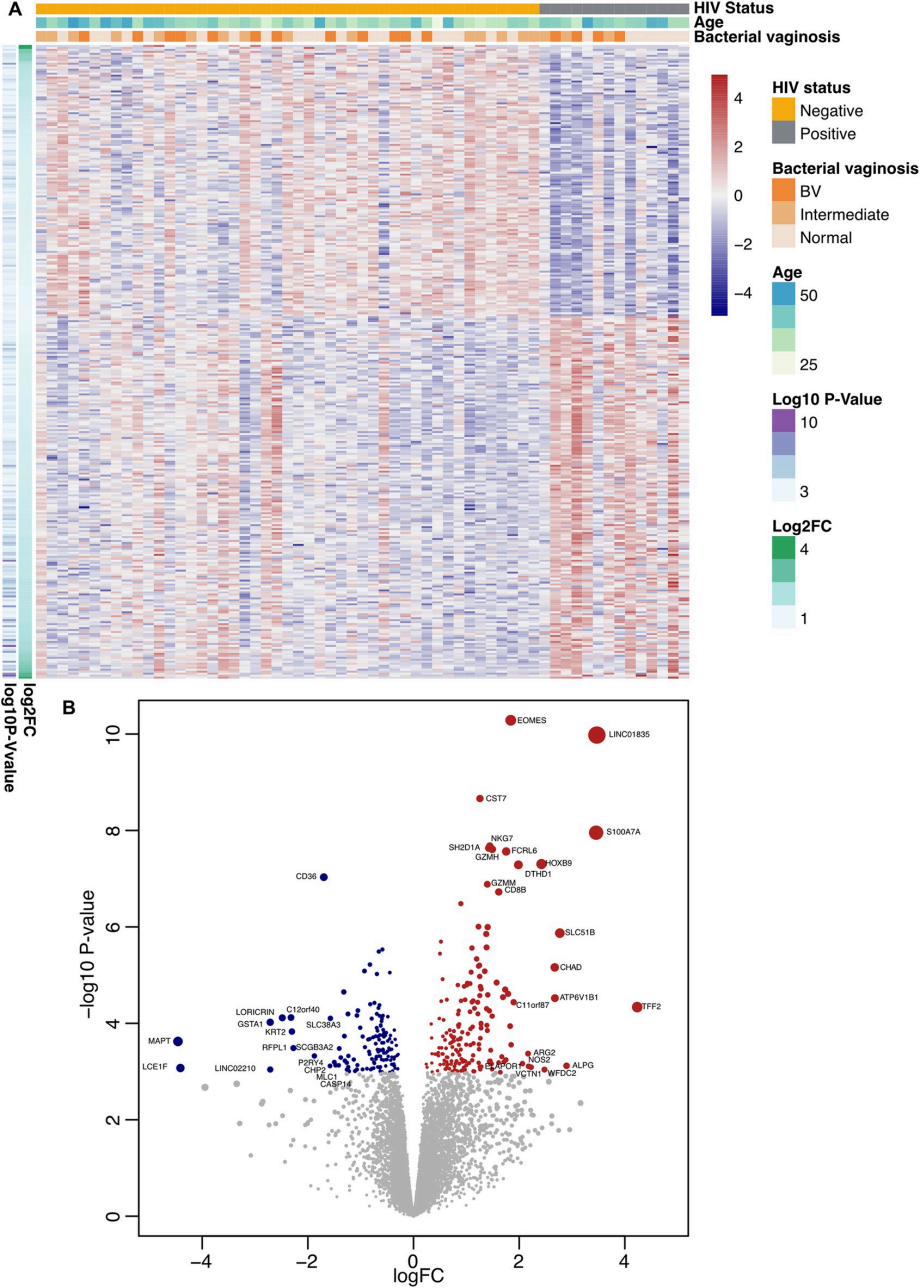
Hierarchical clustering of DEGs illustrates the distinction between samples obtained from HIV^+^FSWs and HIV^–^FSWs. (A) Heatmap displaying all 320 DEGs (FDR-adjusted P < 0.05) identified in HIV^+^FSWs (n = 14) compared to HIV^–^FSWs (n = 47). Each sample is represented by a vertical column, and each gene by a horizontal row. Gene expression values are standardized (z-score) to a mean of 0 and a standard deviation of 1. Blue indicates below-average expression and red indicates above-average expression. Samples from HIV^+^FSWs are gray and those from HIV^–^FSWs are orange. The horizontal bars below the groups represent age and bacterial vaginosis (BV) diagnosis, ranging from normal (0) to intermediate (1) to BV (2), as defined by the Nugent score. (B) Volcano plot illustrating all genes in the dataset, with log_2_FC on the *x*-axis and -log_10_ (*P*-value) on the *y*-axis; each gene is represented by a dot. DEGs upregulated in HIV^+^FSWs are red and DEGs downregulated in HIV^+^FSWs are blue. DEG: Differentially expressed gene. FDR: False discovery rate. FSW: Female sex worker. Log_2_FC: log_2_ fold-change.

Hierarchical clustering analysis of the DEGs demonstrated a distinct separation between the two study groups (**Fig 1A**). Principal component analysis (PCA) of the DEGs revealed no distinct separations between HIV^+^FSWs and HIV^-^FSWs along the first two principal components (**S1 Fig**).

To further elucidate the molecular mechanisms underlying the impact of chronic HIV infection on the cervical mucosa, DEGs were stratified based on their expression patterns between the two study groups. The top 15 most up- and downregulated genes, as determined by fold change (FC), were identified (**Fig 1B** and **Tables 2 and 3**). Among the upregulated DEGs, *TFF2*, a gene upregulated during inflammatory processes and implicated in mucosal tissue repair [29], exhibited the highest FC (log_2_FC = 4.2, FDR-adj *P* = 1.1E^-2^). The remaining top 15 upregulated genes were associated with diverse functions. While inflammation was the predominant function (*S100A7A*, *ALPG*, *WFDC2*, *VTCN1*, *NOS2* and *AGR2*), other genes were involved in metabolism, apoptosis and transport (**Fig 1B and Table 2**). Furthermore, several upregulated genes beyond the top 15 were linked to interferon (IFN) signaling and CD8^+^ T cell activation, effector functions, and exhaustion (**S1 Table**).

**Table 2:** *The top 15 upregulated genes according to log*_*2*_ *fold change between the HIV*^*+*^*FSW and HIV*^*-*^*FSW*.

HGNC ID	Gene name	Log_2_FC	FDR adjusted *P*-Value	Immuno-regulatory
*TFF2*	Trefoil factor 2	4.2	1.1E^-2^	
*LINC01835*	C-type lectin domain family 4 member O, pseudogene	3.5	8.1E^-7^	
*S100A7A*	S100 calcium binding protein A7A	3.5	4.3 E^-5^	X
*ALPG*	Alkaline phosphatase, germ cell	3.2	4.4E^-2^	X
*SLC51B*	Solute carrier family 51 subunit beta	2.9	1.2E^-3^	
*ATP6V1B1*	ATPase H+ transporting V1 subunit B1	2.7	9.1E^-3^	
*CHAD*	Chondroadherin	2.7	3.7E^-3^	
*WFDC2*	WAP four-disulfide core domain 2	2.5	4.6E^-2^	X
*HOXB9*	Homeobox B9	2.4	8.0E^-5^	
*VTCN1*	V-Set domain containing T cell activation inhibitor 1	2.2	4.5E^-2^	X
*NOS2*	Nitric oxide synthase	2.2	4.4E^-2^	X
*AGR2*	Anterior gradient 2, protein disulphide isomerase family member	2.2	3.5E^-2^	X
*ELAPOR 1*	Endosome-lysosome associated apoptosis and autophagy regulator 1	2.1	4.3E^-2^	
*DTHD1*	Death domain containing 1	2.0	8.0E^-5^	
*C11orf87*	Chromosome 11 open reading frame 87	1.9	9.6E^-3^	

HGNC: HUGO Gene Nomenclature Committee. Log_2_FC: Log_2_ fold change. FDR: False discovery rate. Immunoregulatory: involved in immunological processes according to published literature. FSW: Female sex worker.

Conversely, the top 15 downregulated genes exhibited a higher degree of homogeneity. The most significantly downregulated DEG in HIV^+^FSWs was *MAPT* (log_2_FC = –4.5, FDR-adj *P* = 2.5E^–2^), a gene known to enhance microtubule stabilization within intestinal epithelial cells [30]. Consistently, 7 of the top 15 downregulated genes in HIV^+^FSWs were associated with epithelial barrier function and differentiation (*MAPT*, *LCE1F*, *GSTA1*, *LORICRIN*, *KRT2*, *SCGB3A2*, and *CASP14)*, aligning with observations in other inflammatory conditions [28,31,32] (**Fig 1B** and **Table** 3. Re-analysis of the RNA-sequencing data, excluding two HIV^+^FSWs using DMPA, significantly reduced the number of identified DEGs. However, the upregulation of CD8^+^ T cell-related genes was largely retained, along with some IFN-related genes. The downregulated genes were fewer and did not indicate any specific function (**S2 Table**).

**Table 3:** The top 15 downregulated genes according to log_2_ fold change between the HIV^+^FSW and HIV^-^FSW.

HGNC ID	Gene name	Log_2_FC	*P*-Value	Epithelial barrier
*MAPT*	Microtubule associated protein tau	-4.5	2.5E^-2^	X
*LCE1F*	Late cornified envelope 1F	-4.4	4.6E^-2^	X
*GSTA1*	Glutathione S-transferase alpha 1	-2.7	1.7E^-2^	X
*LINC02210*	Long intergenic non-protein coding RNA 2210	-2.7	4.7E^-2^	
*LORICRIN*	Loricrin cornified envelope precursor protein	-2.5	1.5E^-2^	X
*C12orf40*	Chromosome 12 open reading frame 40	-2.3	1.5E^-2^	
*KRT2*	Keratin 2	-2.3	2.1E^-2^	X
*SCGB3A2*	Secretoglobin family 3A member 2	-2.3	3.1E^-2^	X
*RFPL1*	Ret Finger protein like 1	-1.9	3.7E^-2^	
*CD36*	CD36 molecule	-1.7	1.3E^-4^	
*P2RY4*	Pyrimidinergic receptor P2Y4	-1.6	4.4E^-4^	
*SLC38A3*	Solute carrier family member 38 member 3	-1.6	1.5E^-2^	
*CASP14*	Caspase 14	-1.5	4.1E^-2^	X
*CHP2*	Calcineurin like EF-hand protein 2	-1.5	4.4E^-2^	
*MLC1*	Modulator of VRAC current 1	-1.4	4.4E^-2^	

HGNC: HUGO Gene Nomenclature Committee. Log_2_FC: Log_2_ fold change. FDR: False discovery rate. Epithelial barrier: involved in the structure or function of the epithelial barrier according to published literature. FSW: Female sex worker.

Taken together, specific genes associated with immune activation were upregulated in HIV^+^FSWs compared to HIV^–^FSWs, while genes involved in epithelial barrier function and differentiation were downregulated. This suggests that chronic HIV infection significantly impacts gene regulation in the FGT, potentially influencing mucosal immune responses and epithelial integrity.

### Functional enrichment analysis revealed an HIV-associated antiviral response and decreased epithelial barrier integrity

**Fig 2:**
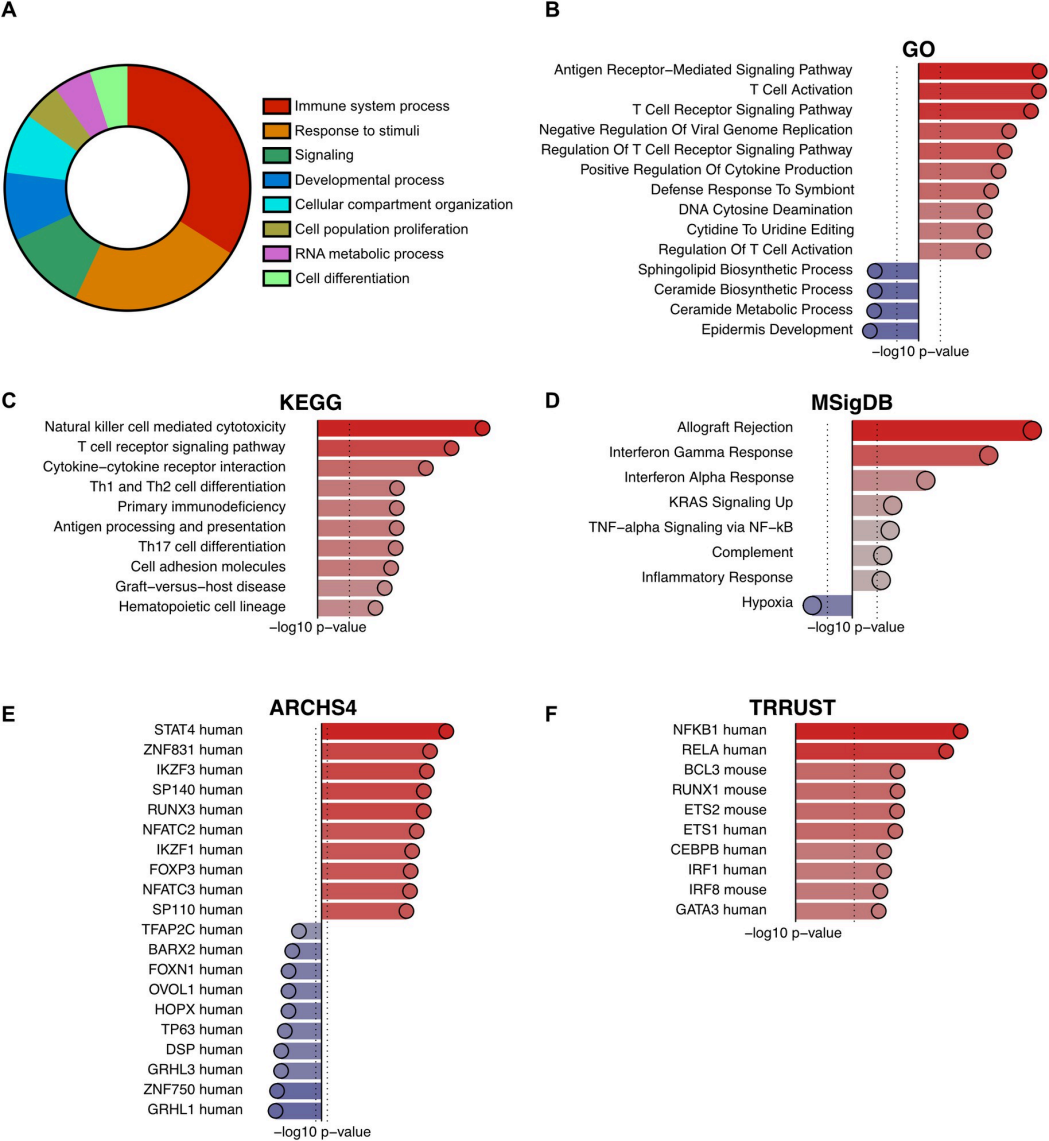
FEA reveals altered pathways associated with immune regulation and epithelial barrier function in HIV^+^FSWs. (A–D) Genes with an FDR-adjusted P < 0.1 identified in HIV^+^FSWs (n = 14) compared to HIV^–^FSWs (n = 47) were subjected to molecular pathway enrichment analysis using the following databases: (A) GO slim, demonstrating major pathways containing at least five distinct enriched pathways, (B) GO, (C) KEGG, and (D) MSigDB. (E) and (F) Transcription factors upregulated and downregulated in HIV^+^FSWs compared to HIV^–^FSWs were identified from DEGs using the (E) TRRUST and (F) ARCHS4 databases. The most significantly upregulated (red) and downregulated (blue) pathways and transcription factors identified were sorted by -log_10_ *P*-value and displayed on the *x*-axis. The dotted vertical line represents a nominal *P* = 0.01. FDR: False discovery rate. FSW: female sex worker. Log_2_FC: log_2_ fold-change.

To obtain comprehensive insight into the functional implications and altered biological pathways associated with chronic HIV infection, Functional Enrichment Analysis (FEA) was conducted on the gene expression data with a cut-off of FDR-adj P-value < 0.1 for genes to be included in the analysis as described [33]. Initially, the gene expression data was evaluated using the Gene Ontology (GO) database [34–36], resulting in the identification of 87 significantly enriched pathways (FDR-adj P-value < 0.05). The GO pathways were then summarized using GO slim focusing on high level categories to highlight the major altered biological processes linked to chronic HIV infection revealing a predominant enrichment of major pathways involved in immunomodulatory processes, encompassing “response to stimuli,” “immune system processes,” and “signaling.” (**Fig 2A** and **S3 Table**).

Subsequently, to provide a more in-depth understanding of the gene expression, FEA was performed using GO, the Kyoto Encyclopedia of Genes and Genomes (KEGG) [37–39] and the Molecular Signature Database (MSigDB) [40,41]. Consistent with the GO slim analysis, the combined FEA analysis underscored a robust immunological response. Notably, pathways involved in cytokine signaling mediated by both type 1 and 2 IFNs and tumor necrosis factor (TNF) α, as well as T cell activation and signaling, were enriched in HIV^+^FSWs. Additionally, pathways associated with “primary immunodeficiency”, “defense against virus” and “Human Immunodeficiency virus 1 infection” were evident in the HIV^+^FSWs (**Fig 2B–2D** and **S4 Table**). Correspondingly, an upregulation of pathways associated with other leukocyte populations, including NK cells and B cells, was evident, indicating a broader immune cell activation (**Fig 2C and S4 Table**).

In contrast, pathways significantly downregulated in HIV^+^FSWs included “Epidermis development” as well as metabolic and biosynthetic processes (**Fig 2B and S4 Table**).

Transcription factor analysis was conducted using All RNA-seq and ChIP-seq Sample and Signature Search (ARCHS4) [42] and Transcriptional Regulatory Relationships Unraveled by Sentence-based Text mining (TRRUST) [43] to elucidate the regulatory networks underlying the gene expression data. Both TRRUST and ARCHS4 predominantly identified upregulation of immunoregulatory transcription factors in HIV^+^FSWs. Notably, ARCHS4 highlighted *IKZF3* and *IKZF1* among the most upregulated transcription factor in HIV^+^FSWs which has been implicated in the regulation of lymphocyte differentiation and promoting HIV persistence [44–46]. Additionally, ARCHS4 identified an upregulation of *IRF1–5* and *IRF7–9*, further implicating IFN signaling as a central factor in immune activation. TRRUST analysis indicated significant upregulation of *NFKB1* (p65), *RELA*, *IRF1* and *IRF8* in HIV^+^FSWs, all of which are involved in IFN signaling (**Fig 2E and 2F and S5 Table**).

Furthermore, ARCHS4 revealed a significant downregulation of transcription factors associated with epithelial barrier maintenance, including *ZNF750*, *GRHL1* and *DSP*, the latter two of which are important regulators of the desmosome [47,48], further implicating a specific disruption of desmosomes. Additionally, downregulation of *FOXN1* was observed, which has been linked to immunodeficiency [49] (**Fig 2E and 2F and S5 Table**).

Taken together, these results highlight an HIV-associated pro-inflammatory environment within the cervix, primarily mediated through IFN signaling and T cell activation, indicating a sustained antiviral response and a decreased epithelial integrity and altered metabolic regulation.

### Plasma viral load correlated with sustained mucosal anti-viral immune responses

**Fig 3:**
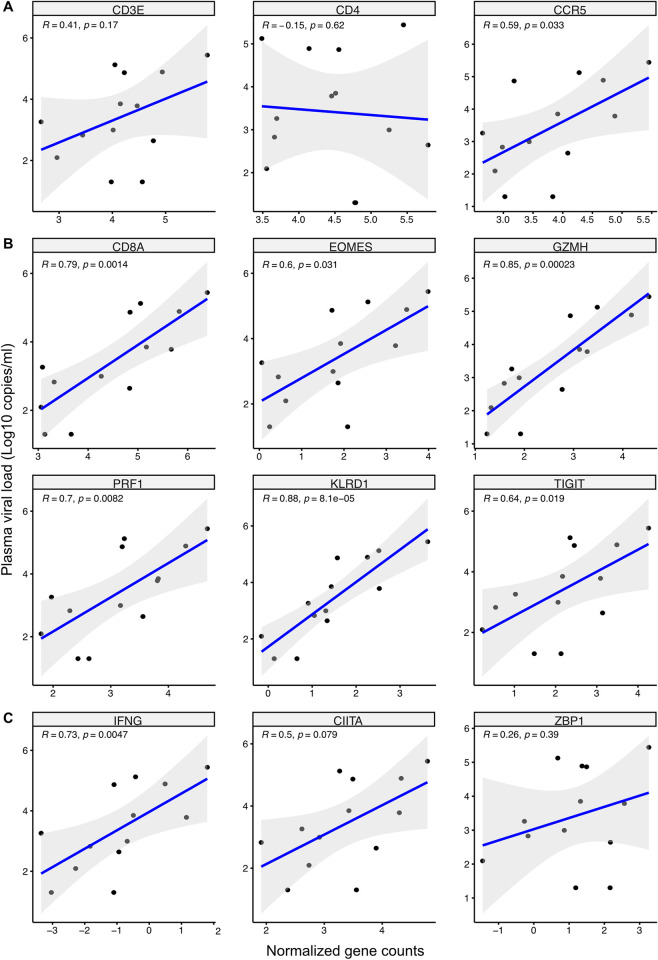
Plasma viral load positively correlated with genes involved in T cell signaling and IFN response. Correlation analysis of plasma viral load and identified upregulated genes within HIV^+^FSWs involved in T cell activity and IFN signaling. (A) General T cells related genes *CD3E*, *CD4* and *CCR5*. (B) CD8^+^ T cell related genes *CD8A EOMES*, *GZMH*, *PRF1*, *KLRD1* and *TIGIT*. (C) IFN related genes *IFNG*, *CIITA* and *ZBP1*. The graphs illustrate Spearman correlations between gene count and plasma viral load (counts/ml) with a linear regression line, the 95% confidence interval, and all individual data points. The log_10_ plasma viral load is shown on the y axis and the normalized gene count is shown on the x axis. P<0.05 was considered significant. IFN; Interferon.

To further corroborate the RNA-sequencing data, the correlation between plasma viral load and selected genes was assessed within the HIV^+^FSWs. Due to the strong induction of T cells and IFN signaling, genes were selected based on their functional relevance in HIV infection and within these key pathways (**Fig 2 and S1 and S4 Tables**). Assessing the general T cell markers *CD3E* and *CD4* revealed no correlation. A positive correlation was however observed between viral load and the HIV co-receptor *CCR5* (**Fig 3A**). Assessing CD8^+^ T cell related genes revealed a positive correlation between viral load and expression of *CD8A*. A significant positive correlation could be observed for the transcription factor *EOMES* associated with dysfunctional CD8^+^ T cells during chronic HIV infection [50]. Assessing cytotoxic properties revealed a positive correlation for both *GZMH* and *PRF1*, as well as *KLRD1*, primarily expressed on cytotoxic T and NK cells. Additionally, the exhaustion marker *TIGIT* also demonstrated a positive correlation with viral load (**Fig 3B**). The IFN-related genes demonstrated a positive correlation between viral load and expression of *IFNG*, but not of *CIITA* or *ZBP1* (**Fig 3C**). *TFF2* and *S100A7A* were further selected based on their FC, however no correlation could be observed for either of them (**S2A Fig**). Finally, downregulated genes involved in epithelial stability were selected based on FC and included *LCE1F*, *LORICRIN* and *KRT2*. However, no correlation between viral load and gene expression could be observed (**S2B Fig**). Collectively, these results indicate a potential causal link between plasma viral load and cervical inflammation mediated by CD8^+^ T cell activation and IFN signaling.

### Development of a bioimage analysis workflow for evaluation of epithelial integrity and spatial distribution of epithelial junction proteins

To complement and validate the transcriptional data, we established a high-throughput quantitative bioimage analysis workflow to evaluate ectocervical epithelial stability in HIV^+^FSWs by examining a panel of epithelial junction proteins (EJPs). Our *in situ* immunofluorescence staining panel consisted of the transmembrane adherent junction protein E-cadherin, the desmosomal protein desmoglein-1 (DSG1), the tight junction protein claudin 1, and the scaffolding protein zonula occludens 1 (ZO1), which anchors tight junctions to the actin cytoskeleton. The EJPs collectively form net-like structures that contribute to cervical epithelial stability and prevent paracellular penetration of incoming pathogens.

**Fig 4:**
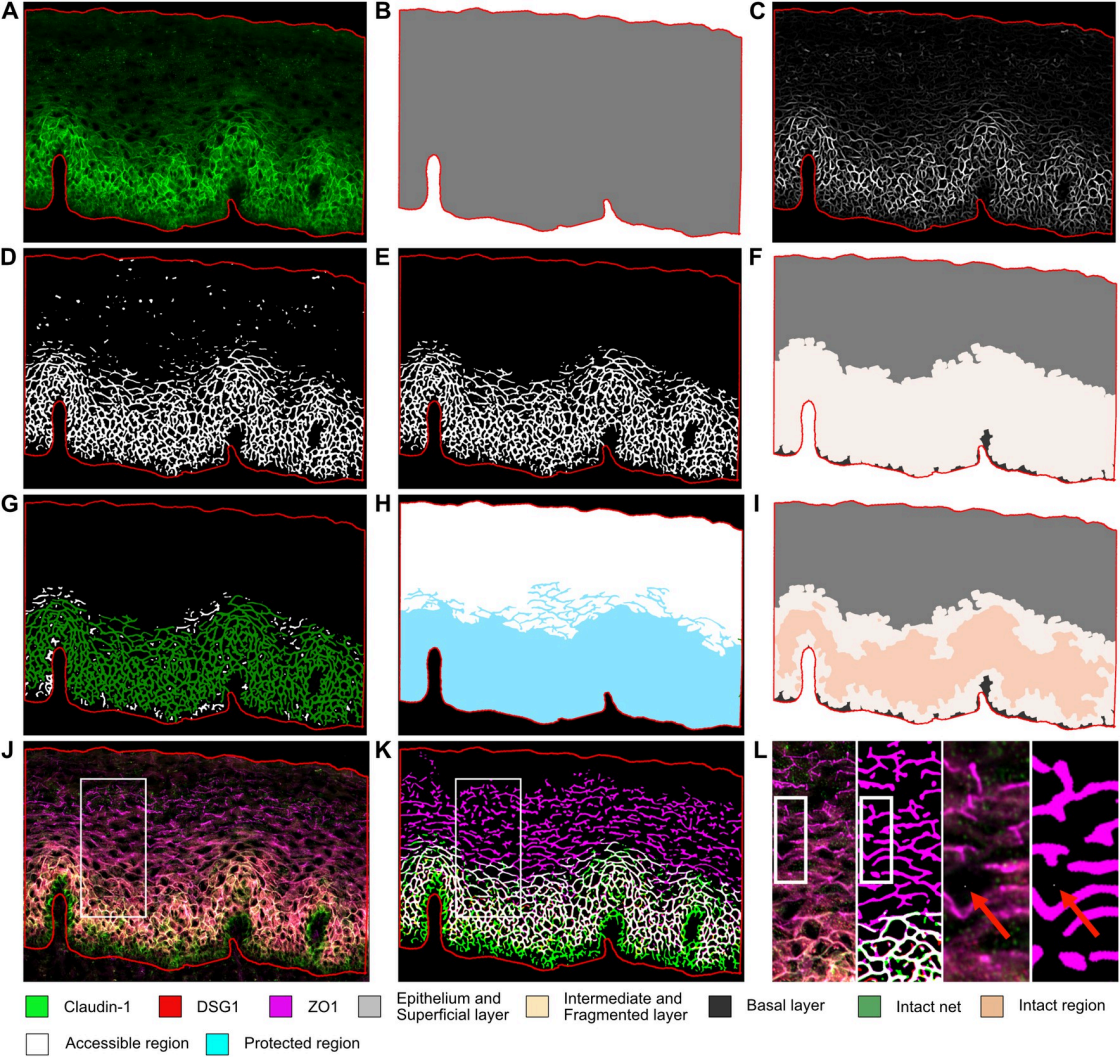
Workflow for bioimaging analysis of the ectocervical epithelial compartment. Schematic representation of the bioimage analysis developed to evaluate ectocervical epithelial integrity. The epithelial compartment is outlined in red in all images. (A–K) Bioimage analysis of individual epithelial junction proteins (EJPs). (A) Fluorescent image of the ectocervical mucosa stained for claudin-1. (B) The outlined epithelial compartment used for subsequent analyses. (C) Curve-linear enhancement to facilitate segmentation. (D) Identification of the net-like protein structure. (E) Size exclusion to remove artifacts. (F) Division of the epithelial compartment into superficial (gray), intermediate (IM; containing the junction protein) (light beige), and basal layer (dark gray) by expanding the identified net-like structure of the protein strands. (G) Classification of the identified net-like structure as intact (green) or fragmented (white) by size exclusion. (H) Flooding watershed transformation from the cervicovaginal border towards the basal membrane to generate a theoretical protected (cyan) and accessible region (white) for an incoming virus based on the intact net. (I) Further division of the IM layer into intact (beige) and fragmented (light beige) regions based on the intact net-like structure. (J) Bioimaging analysis of the combined EJPs claudin-1 (green), DSG1 (red), and ZO1 (magenta). (K) Merging of the identified net-like structures of claudin-1, DSG1, and ZO1 to enable a combined analysis, with the analysis pipeline described in (A–I) applied to the combined layer. (L) Zoom in of the highlighted areas in J-K to illustrate the size of a pixel (white dot) as indicated by the red arrow. Brightness and contrast were enhanced in the original images in (A) and (J) for visualization purposes. DSG1: desmoglein-1. ZO1: zonula occludens 1. IM: intermediate layer.

Using this workflow, we first measured the mean fluorescence intensity (MFI) of each junctional protein, which served as a proxy for protein expression. However, as the MFI only provides information regarding protein expression and not spatial distribution or integrity of the EJPs, the workflow was further developed to identify the epithelium and the net-like structure of each EJP (**Fig 4A–4E**). The epithelium was stratified into layers based on the expression pattern of the EJPs, distinguishing superficial and basal layers devoid of EJP expression from an intermediate (IM) layer marked by EJP expression (**Fig 4F**). To investigate the association between chronic HIV infection and epithelial integrity, we classified the identified net-like structure of the EJPs as intact or fragmented, depending on the connectivity of the protein strands (**Fig 4G**). Subsequently, we generated a theoretical model of epithelial accessibility for an incoming or outgoing virus by digitally flooding the epithelium from the cervicovaginal border towards the basal membrane, where the intact net acted as the primary barrier, thus generating an accessible and a protected region (**Fig 4H**). Furthermore, we utilized the intact net to assess epithelial stability by dividing the IM layer into intact and fragmented regions (**Fig 4I).** While the bioimage analysis was performed individually for all markers, an extension of the workflow was developed. This extension allowed for the merging of the identified protein strands of claudin-1, DSG1, and ZO1 for a combined analysis, thereby providing a more comprehensive assessment of epithelial integrity (**Fig 4J–4K**). Any break larger than one pixel (0.325μm) was considered a sufficient break to classify the net as fragmented and allow for viral transmigration (**Fig 4L**).

### HIV^+^FSWs showed signs of ectocervical epithelial disruption primarily driven by DSG1 and claudin-1

**Fig 5:**
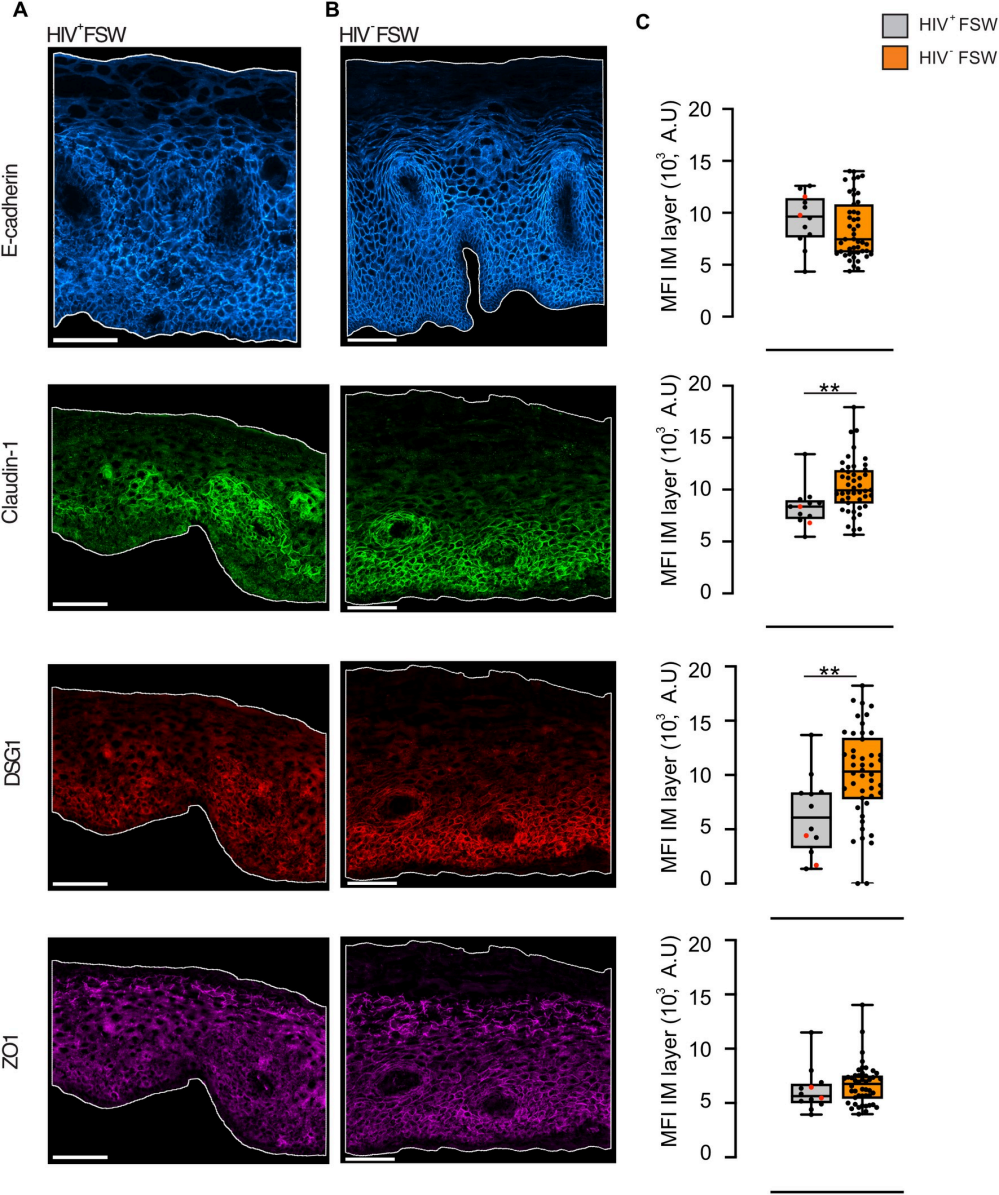
Expression of claudin-1 and DSG1 is reduced in HIV^+^FSWs. Representative images of E-cadherin (blue), claudin-1 (green), DSG1 (red), and ZO1 (magenta) in ectocervical tissue from (A) HIV^+^FSW (n = 12) and (B) HIV^-^FSW (n = 47). Brightness and contrast were enhanced in all images for visualization purposes. Scale bars represent 100 μm, and the epithelium is outlined in white. (C) Boxplots illustrating the MFI within the IM layer of E-cadherin, claudin-1, DSG1, and ZO1. Boxes represent the median and IQR, while whiskers indicate the full range. HIV^+^FSWs using DMPA have been highlighted in red. Statistical analysis was conducted using the Mann–Whitney U test, with significance set at *P*<0.05. **, *P*<0.01. FSW: female sex worker. DSG1: desmoglein-1. ZO1: zonula occludens 1. MFI: mean fluorescence intensity. IM: intermediate layer. IQR: interquartile range. DMPA: Depot medroxyprogesterone acetate.

Between the two study groups, the total assessed epithelial tissue areas were similar and no significant difference in epithelial height was observed. The MFI of each junctional protein was measured and the HIV^+^FSWs group, compared to the HIV^–^FSW group, exhibited significantly decreased expression of both claudin-1 and DSG1 (*P* = 0.005, *P* = 0.004, respectively) within the IM layer (**Fig 5**). When analyzing the entire epithelial compartment, only a significant decrease in DSG1 expression (*P* = 0.03) persisted among HIV^+^FSWs compared to the HIV^–^FSWs (**S3 Fig**).

**Fig 6:**
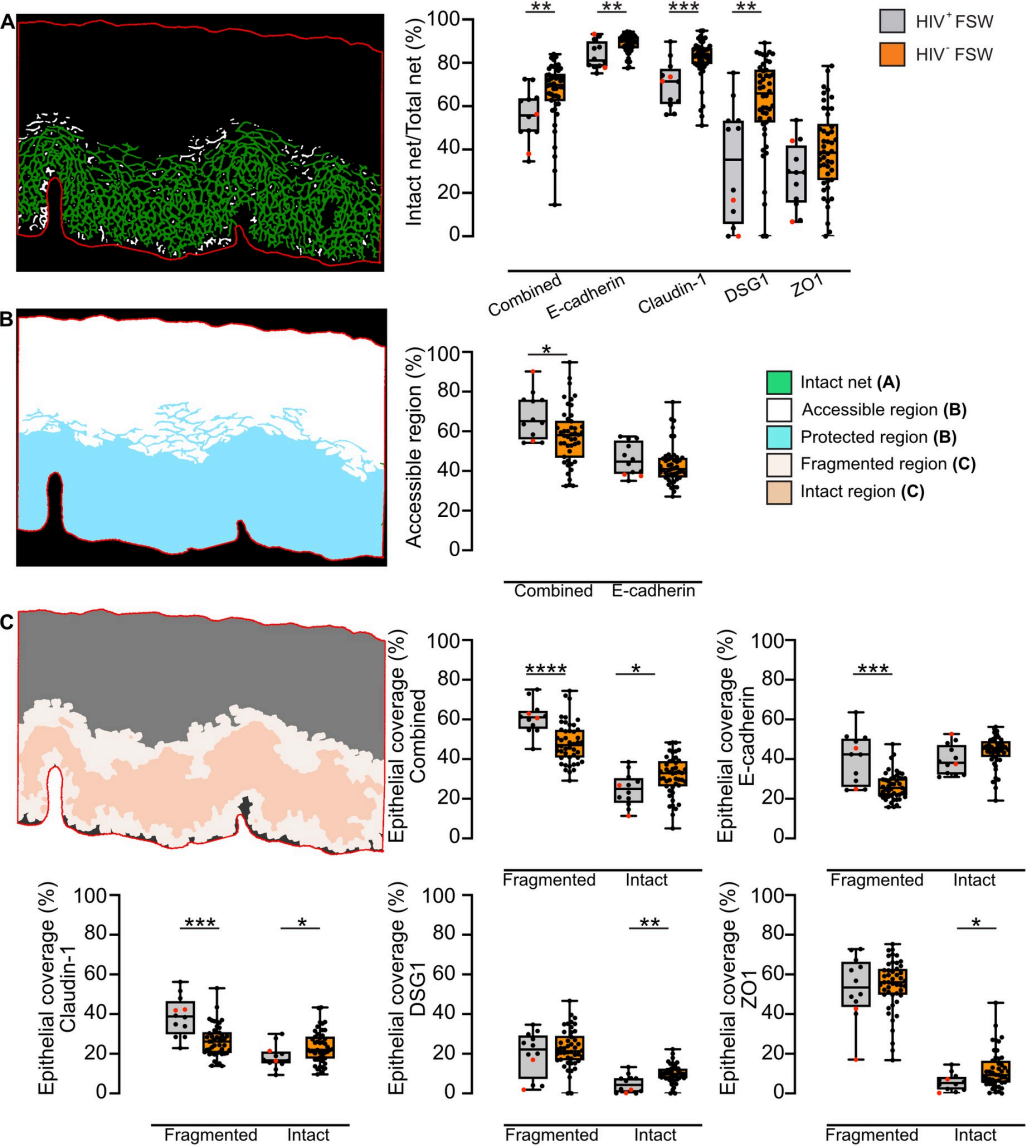
The ectocervical epithelium is disrupted in HIV^+^FSWs. (A) Left: Representative image showing the net-like structure formed by the segmented junction protein claudin-1, with intact net in green and fragmented net in white. Right: Boxplots indicating the percentage of the total net considered intact for combined analysis of claudin-1, DSG1, and ZO1, as well as for individual analysis of E-cadherin, claudin-1, DSG1, and ZO1. Samples from HIV^+^FSWs (n = 12) are gray, and samples from HIV^–^FSWs (combined analysis, claudin-1, DSG1, and ZO1; n = 46, E-cadherin; n = 47) are orange. (B) Left: Representative image demonstrating the theoretical accessible region (white) and protected region (blue) based on the combined analysis. Right: Boxplots showing the theoretical epithelial accessibility of the combined analysis and E-cadherin. (C) Left: Representative image illustrating the fragmented (light beige) and intact (dark beige) regions within the IM layer based on claudin-1 staining. The superficial and basal layers are outlined in gray and dark gray, respectively. Right: Boxplots indicating the epithelial coverage of the identified fragmented and intact regions for the respective junction proteins. Boxes represent the median and IQR, while whiskers indicate the full range. HIV^+^FSWs using DMPA have been highlighted in red. Statistical analysis was conducted using the Mann–Whitney U test, with significance set at *P*<0.05. *, *P*<0.05; **, *P*<0.01; ***, *P*<0.001; ****, *P*<0.0001. FSW: female sex worker. DSG1: desmoglein 1. ZO1: zonula occludens 1. IM: intermediate layer. IQR: interquartile range. DMPA: Depot medroxyprogesterone acetate.

Given that successful infection by several viruses, including HPV requires a disrupted epithelium [51,52], epithelial integrity was assessed next as MFI only provides an indication for protein expression levels. Compared to HIV^–^FSWs, HIV^+^FSWs displayed a significantly lower proportion of intact net (out of total net) for the combined analysis (*P* = 0.002) and individually for E-cadherin, claudin-1, and DSG1 (*P* = 0.007, *P* = 0.0008, and *P* = 0.001, respectively) (**Fig 6A**). Subsequently, the theoretical epithelial accessibility for an incoming viral particle was assessed. Results from the combined analysis demonstrated a significantly increased epithelial accessibility in HIV^+^FSWs compared to HIV^–^FSWs (*P* = 0.03), although this was not observed for E-cadherin (**Fig 6B**). Finally, the stability of the epithelium was analyzed. Compared to HIV^–^FSWs, HIV^+^FSWs displayed a significantly larger epithelial coverage of the fragmented junctional layer for the combined markers (*P<*0.0001) and individually for E-cadherin (*P* = 0.0004) and claudin-1 (*P* = 0.0001), but not for DSG1 or ZO1. Accordingly, a significantly smaller epithelial coverage of the intact regions was observed within the HIV^+^FSWs based on the combined markers (*P* = 0.03) and individually for claudin-1 (*P* = 0.03), DSG1 (*P* = 0.002), and ZO1 (*P* = 0.03), but not E-cadherin (**Fig 6C**) Collectively, these results demonstrate that in addition to a decreased expression of DSG and claudin-1, HIV^+^FSWs also demonstrate indications of a decreased stability of all EJPs.

**Fig 7:**
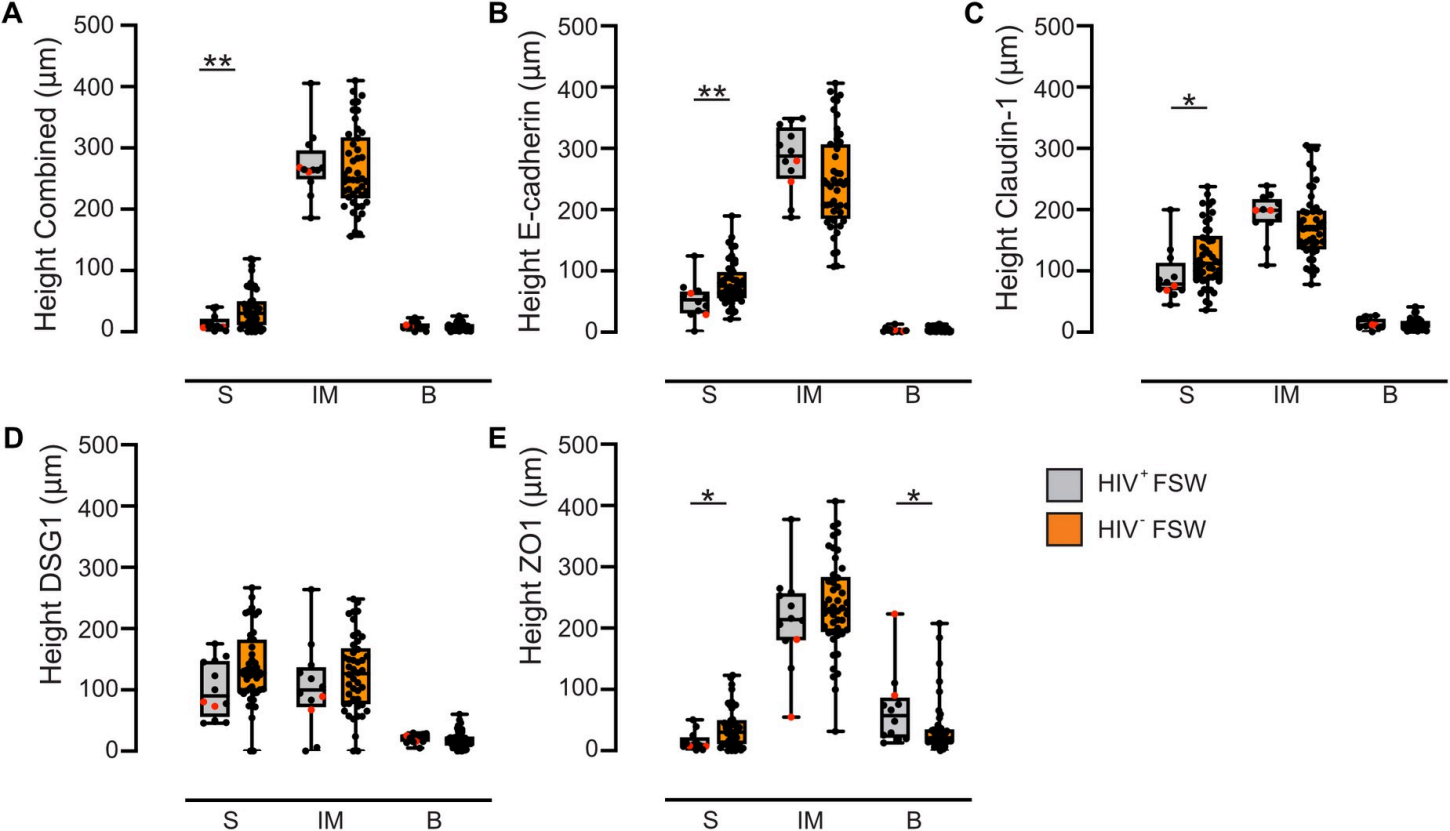
The ectocervical superficial layer is thinned in HIV^+^FSWs. Boxplots demonstrate the height of the three epithelial layers: superficial (S), intermediate (IM), and basal (B) for (A) the combined analysis, (B) E-cadherin, (C) claudin-1, (D) DSG1, and (E) ZO1 in HIV^+^FSWs and HIV^–^FSWs. Samples from HIV^+^FSWs are gray, and samples from HIV^–^FSWs are orange. Boxes represent the median and IQR, while whiskers indicate the full range. HIV^+^FSWs using DMPA have been highlighted in red. Statistical analysis was conducted using the Mann–Whitney U test, with significance set at *P*<0.05. *, *P*<0.05; **, *P*<0.01. FSW: female sex worker. DSG1: desmoglein 1. ZO1: zonula occludens 1. IQR: interquartile range. DMPA: Depot medroxyprogesterone acetate.

The spatial distribution of each EJP was evaluated by measuring the height of the superficial, IM, and basal layers. Based on the combined analysis, the superficial layer was significantly thinner in HIV^+^FSWs than in HIV^–^FSWs (*P* = 0.009), as well as in the individual analyses of E-cadherin, claudin-1, and ZO1 (*P* = 0.006, *P* = 0.03, and *P* = 0.02, respectively), but not DSG1 (**Fig 7**). No significant differences in the height of the IM layer were observed between the study groups based on either the combined analysis or the individual EJPs. Furthermore, HIV^+^FSWs exhibited a significantly thicker basal layer based on ZO1 staining (*P* = 0.02), although this difference was not evident in combined analysis or staining of any of the other EJPs (**Fig 7**). Thus, an altered spatial distribution of E-cadherin, DSG1 and claudin 1 was associated with chronic HIV infection.

Overall, our bioimage analysis data collectively indicate that the HIV^+^FSW group demonstrated signs of epithelial disruption, primarily characterized by the downregulation of claudin-1 and DSG1, with the latter aligning well with the observed epithelial disruption in our transcriptional data.

### Altered spatial distribution of CD4^+^ cells in ectocervical epithelium of HIV^+^FSWs compared to HIV^-^FSWs

We investigated the frequency and spatial distribution of intra-epithelial CD4^+^ cells, given their significance in HIV infection and dissemination. Our analysis revealed no significant difference in CD4^+^ cell frequency between the study groups (**S4A Fig**). Calculations of the average, lower quartile (LQ), and upper quartile (UQ) distances of CD4^+^ cells to the superficial epithelial border showed a significantly shorter LQ distance in HIV^+^FSWs compared to HIV^-^FSWs (*P* = 0.04) (**S4B Fig**). Assessing the correlation between blood and epithelial CD4^+^ cells revealed no significant difference (**S4C Fig**). Therefore, our findings indicate that there was no significant depletion of CD4^+^ cells in the ectocervical epithelium of HIV^+^FSWs, consistent with our previous study [16]. Additionally, CD4^+^ cells in HIV^+^FSWs were more apically located within the ectocervical epithelium compared to HIV^-^FSWs.

## Discussion

This study represents a pioneering effort to investigate the impact of chronic HIV infection on cervical mucosa, utilizing a combination of transcriptional and bioimaging techniques on unique ectocervical tissue samples. Our results unveil significant alterations in gene expression profile and epithelial integrity among HIV-infected women, illuminating the intricate relationship between chronic HIV infection and mucosal immunity.

In our study, HIV^+^FSWs exhibited upregulation of genes associated with immune activation and inflammation, particularly involving T cell activity and IFN signaling which largely correlated with plasma viral load, along with downregulation of genes important for epithelial barrier structure and function. Furthermore, using our novel quantitative bioimage analysis workflow, we observed decreased expression and disruption of selected EJPs, essential for maintaining a robust and protective epithelial compartment, in HIV^+^FSWs. These findings contribute to a better understanding of how immune activation in chronically HIV-infected women affects mucosal tissue compartments and may guide future interventions for FGT-related complications, including STIs and cervical cancer.

Chronic inflammation and immune activation are recognized as hallmarks of chronic HIV infection [3]. The underlying causes of this inflammation remain incompletely understood but are thought to involve intrinsic and extrinsic factors such as altered cytokine signaling, immune cell dysfunction, and microbial translocation [3,7–9]. While cervical immune activation in HIV infection has been documented [12,14,21], a comprehensive characterization of this phenomenon is lacking. To our knowledge, this study represents the first application of RNA-seq to analyze the cervix of chronically HIV-infected women.

The DEGs observed within the FGT of HIV^+^FSWs exhibited a high degree of homogeneity, representing distinct functions. Upregulated genes were overwhelmingly immunoregulatory, consistent with previous observations [12,21], which further largely correlated with plasma viral load, while downregulated genes demonstrated a broader heterogeneity but were overrepresented by genes involved in epithelial barrier structure and function.

Given the cross-sectional study design, we cannot definitively attribute these differences solely to HIV infection and DMPA had a significant impact on gene expression. However, the women lacked concomitant STIs and were rigorously matched for age, time in sex work and being in the follicular stage of the menstrual cycle. No significant differences between the study groups were found for sexual behavior or cervicovaginal microbiome composition at both clinical and molecular levels. This makes chronic HIV infection a likely distinguishing factor. The prevalence of HSV-2 and HPV was however not recorded and cannot be excluded as confounding factors. Our previous study from the same cohort demonstrated an HSV-2 seroprevalence above 94% in both HIV seronegative and HIV seropositive sex [53]. Although the HPV prevalence (HPV DNA) has not been published for the present cohort, a comparable sex worker cohort from the Nairobi area showed a higher HPV prevalence within HIV^+^FSWs compared with HIV^-^FSWs (32% vs 24%, respectively), and can thus not be ruled out as a confounding factor [54].

Fittingly, the top-upregulated gene, *TFF2*, plays a dual role in stabilizing the gastric mucus barrier layer and promoting mucosal healing, potentially in response to the observed epithelial disruption [29], while overexpression and ectopic expression of *TFF2* are implicated in various pathological conditions, particularly those involving chronic inflammation [55, 56], which aligns with our observations. Moreover, *TFF2* expression could induce cytokine secretion, further exacerbating the observed inflammatory state [57]. However, as no correlation could be observed comparing expression of *TFF2* with viral load, it is possible that the alterations are a result of the initial damage caused by HIV, or as an indirect effect of the ongoing chronic inflammation [18,58].

Our FEA revealed a significant increase in cytokine-mediated signaling in HIV^+^FSWs. These cytokines included TNFα, interleukins and IFNs. IFNs, primarily secreted during viral infections, induce an antiviral state that inhibits replication and enhances immune responses [59]. The observed IFN response, coupled with pathways involving antiviral defense and immunodeficiency, suggests that chronic immune activation in HIV-infected women is characterized by a sustained antiviral state. The persistent HIV-associated IFN signaling could exacerbate disease progression and contribute to HIV-mediated systemic chronic immune activation [7,60]. Thus, our identification of sustained IFN signaling in the cervix aligns with systemic findings, implying it is a probable driver of chronic cervical immune activation.

Furthermore, our analysis unveiled an upregulation of pathways associated with several leukocyte populations, indicating prolonged activation or enhanced cervical homing. T cells emerged as the predominant induced leukocyte population, with pathways related to both activation and differentiation. For example, several DEGs associated with CD8^+^ T cells, including genes such as *GZMH/K/M* that encode effector functions, along with indicators of exhaustion including *TIGIT*, *TOX* and *LAG3*, were upregulated. These findings, along with the noted increase in pathways linked to immunodeficiency and the predicted decreased *FOXN1* expression, which is associated with T cell immunodeficiency [49], imply that dysregulated T cell responses or an altered balance of T cell subsets are probable contributors to HIV-associated chronic cervical inflammation [12,21,61].

Given the crucial role of IFNs in functional T cell responses, these observations are likely intrinsically linked with the observed sustained IFN signaling. Additionally, the transcription factor *IKZF3*, predicted to be a highly upregulated transcription factor within HIV^+^FSWs, promotes survival of HIV-infected cells, with heightened expression within both latent and activated HIV-infected T cells [45]. Consistent with our present findings, we previously reported an increased frequency of CD8^+^ T cells in the ectocervical mucosa of a similar cohort, further implicating dysregulated CD8^+^ T cell responses in HIV-mediated cervical inflammation [61].

Our results revealed a notable correlation between plasma viral load and transcriptional markers of CD8^+^ T cell activity. More specifically, we observed that the transcription factor *EOMES*, which has been linked to an exhausted phenotype of CD8^+^ T cells [50], correlates with viral load, as did the exhaustion marker *TIGIT*. A strong correlation was also observed with both *GZMH* and *PRF1*, indicating a cytotoxic phenotype. However, since *KLRD1*, a potential immunoregulator of cytotoxicity, also demonstrated a strong correlation, it may modulate this cytotoxic response [62]. Although these are compelling indications of a dysregulated CD8^+^ T cell response, it is also possible that an aberrant NK cell activation could contribute to these observations. Finally, ectocervical expression of *IFNG* showed a strong positive correlation with viral load in line with persistently elevated levels observed systemically [63]. While these results do not prove causality between chronic HIV infection and sustained cervical immune activation, they indicate that chronic HIV may play a key role in affecting cervical mucosa similarly to its systemic impact.

Previous studies underscore the crucial role of an intact epithelium in preventing STIs [51,52]. Experimental models have demonstrated HIV-mediated epithelial disruption, downregulation of several EJPs, and reduced transepithelial resistance, attributed to viral interaction and induction of cytokines such as TNFα and IFNs [18,58,64]. Although cytokine expression at the protein level was not measured within the ectocervical tissue, an HIV-associated upregulation of immunoregulatory genes and pathways involved in cytokine signaling, including IFNs and TNFα, was observed. This suggests their potential contribution to the observed epithelial disruption.

Our transcriptomic analysis unveiled significant downregulation of genes encoding key epithelial barrier proteins responsible for maintaining stability and connecting adjacent cells [65]. FEA further confirmed the downregulation of epidermis development, providing evidence for HIV-associated epithelial disruption within the ectocervix during chronic HIV infection. These findings are supported by the predicted HIV-associated downregulation of transcription factors involved in the maintenance and function of the epithelial barrier, including *ZNF750* and *GRHL1* [65,66]. The decreased expression of epithelial genes did not correlate with plasma viral load. It is however possible that the observed cervical immune induction, which correlated with viral load, is influencing epithelial stability as an indirect effect of the chronic infection [18,58,64]. Similar to our discussion for *TFF2*, it is also possible that the disruption is a result of the initial infection which does not heal due to the chronic infection.

Besides, our RNA-seq findings were validated through quantitative bioimaging analysis, unveiling pronounced indications of compromised epithelial integrity and stability alongside heightened viral accessibility, as evidenced by alterations in EJP expression. The evaluated panel was chosen to encompass the major families of epithelial junctional proteins, including adherens junctions, tight junctions and desmosomes. While this selection may not perfectly mirror the epithelial disruption observed in the RNA-sequencing data, it aimed to provide a comprehensive representation of the epithelium as a whole. Specifically, a decreased DSG1 and claudin-1 expression and increased fragmentation of all EJPs were observed, both of which could indicate a more accessible epithelium for viruses, whether incoming or outgoing. These changes could contribute to the increased prevalence of other STIs in HIV-infected individuals [24–26], as well as the shedding of HIV particles [51,52]. Notably, DSG1 emerged as one of the most affected EJPs, exhibiting disruption and decreased expression consistent with our transcriptional data. Moreover, *ex vivo* analysis confirmed heightened epithelial fragmentation during chronic HIV infection, consistent with prior research following acute HIV challenge [18,67]. Of note, claudin-1 and DSG1 exhibited the most severe fragmentation, while ZO-1 and E-cadherin were comparatively less affected. Collectively, our integrated findings from both transcriptional and bioimaging analyses of the effects of chronic HIV infection align with previous studies on cervical cell lines and explants, indicating epithelial disruption as a consequence of acute HIV infection [18,58,67,68].

Building upon our previous findings from a similar cohort [16], our bioimaging analysis found no significant HIV-associated differences in CD4^+^ cell abundance within the ectocervical epithelium. These observations, coupled with the upregulation of CD4^+^ T cell-related pathways, suggest a more stable population compared to their significant decrease within the GI tract of HIV-infected individuals [16,69].

Limitations of this study include the use of bulk rather than single-cell RNA-seq and we could thus not distinguish between different cell populations within our RNA-seq dataset. Additionally, the use of bulk RNA-seq does not allow for spatial analysis and we can therefore not determine where in the tissue the observed differences occurred. Previous studies have however, demonstrated that T cells only represent a small fraction of all cells within the ectocervical epithelium, and the majority of the T cells are aggregating around the basal membrane within the ectocervix. Additionally, we have previously demonstrated that roughly 50% of all immune cells within the ectocervix are T cells which support the current observations [16,53,61,70]. The study lacks the distinction between different CD4^+^ cell subpopulations, which could have provided valuable insights. However, previous studies have demonstrated CD4^+^ T cells as the predominant CD4^+^ cell population within the ectocervix, further supporting the notion of a relatively stable cervical population within HIV infected women [16,71]. Staining for HIV particles could have further elucidated our findings, given the presence of viral reservoirs in the cervical mucosa [72], potentially contributing to the observed increase in immunoregulatory genes and pathways. Unfortunately, this study lacks plasma levels of inflammatory markers from these individuals, which could have provided valuable insight into the relationship between systemic and localized immune activation. Similarly, we do not have data on viral load levels within the FGT. However, a previous study from the same cohort demonstrated a positive correlation between viral load in plasma and cervicovaginal fluid, suggesting this correlation is likely present in our study as well [53]. Finally, as none of these women were on anti-retroviral treatment, further studies are needed to assess if the observed immune activation and epithelial disruption is reverted following treatment.

Nevertheless, integrating RNA-seq with bioimaging provided valuable insight into the spatial distribution, expression, and morphological parameters of EJPs within the ectocervix. While our study primarily focused on HIV-associated disruption of the epithelial compartment, our bioimaging workflow holds broader implications in epithelial biology. By addressing these limitations and leveraging the strengths of the study, such as the integration of multiple techniques, our findings can inform novel therapeutics and identify risk factors for acquiring STIs. Developed initially for the stratified squamous epithelium of the ectocervix, our imaging technique is adaptable to similar tissues, promising broader applicability.

In conclusion, our study provides a comprehensive assessment of the impact of chronic HIV infection on cervical tissue in HIV-infected women, employing a combined approach of RNA-seq and bioimaging analysis. We identified notable HIV-associated cervical immune activation, marked by a heightened antiviral state driven by T cell activity and IFN signaling, correlating with plasma viral load. Additionally, our results revealed a simultaneous downregulation of genes critical for epithelial barrier function and structure, supported by FEA and bioimaging analysis. This underscores a potential mechanistic connection between HIV-induced cervical immune activation and compromised epithelial health, elucidating the pathway by which HIV infection could heighten the risk of acquiring other STIs. Moving forward, addressing limitations such as the absence of single-cell RNA-seq analysis and exploring therapeutic interventions based on our mechanistic insights are crucial steps in mitigating HIV-related complications and reducing the burden of STIs.

## Methods

### Ethics statement

This study was performed in accordance with the Helsinki Declaration and was approved by the ethical review boards at the University of Manitoba, (HS15280(B2012:043), the Kenyatta National Hospital University of Nairobi (P224/04/2012, amendment 2017-04-03), and the regional Ethical Review Board in Stockholm (KI:2018:1306–31). Written informed consent was obtained from all participants.

### Study subjects and sample collection

Participants in the study included HIV^+^FSW and HIV^–^FSW recruited from the larger Pumwani Sex Worker Cohort in Kenya. Selection criteria were based on a larger longitudinal study conducted within this cohort [27]. Inclusion criteria comprised FSWs aged 18–50 years, not pregnant or breastfeeding, not menopausal, without prior hysterectomy, and willing to undergo ectocervical biopsy collection. Sampling was aimed at the follicular phase of the menstrual cycle based on self-reported time since last menses. Plasma estradiol and progesterone levels were measured using electrochemiluminescence immunoassays (Roche Diagnostics) with a lower limit of detection for estradiol and progesterone set at 22 pg/ml and 0.05 ng/ml respectively. Participants were required to test negative for *Chlamydia trachomatis*, *Neisseria gonorrhoeae*, *Treponema pallidum* and *Trichomonas vaginalis* infection at the time of enrollment. *Chlamydia trachomatis* and *Neisseria gonorrhoeae* were screened by performing PCR on urine samples using the Roche AMPLICOR kit (Pleasanton, NJ, USA). *Treponema pallidum* was detected by Macro-Vue Rapid Plasma Reagin test (Becton Dickinson, NJ, USA) while saline microscopy was used to detect *Trichomonas vaginalis*. Additionally, bacterial vaginosis was assessed using Nugent score on gram-stained smears. The molecular microbiome composition was evaluated through microbial 16S rRNA sequencing of cervicovaginal lavage [28].

Participants answered a demographic and behavioral questionnaire and were required to abstain from vaginal intercourse for a 2-week period before and after sampling, as well as to undergo regular testing for the presence of prostate-specific antigen. The participants were monetarily compensated for loss of income during this period. HIV^+^FSWs had no history of AIDS-defining illnesses, and none were receiving antiviral therapy, as their CD4^+^ T cell counts exceeded the threshold typically indicating the need for initiation of antiretroviral therapy at the time of study inclusion. Women who tested HIV seronegative at study onset remained seronegative for 3–6 months after study completion.

All participants received counseling on preventing STIs, along with male and female condoms, family planning services, and access to medical care, including HIV treatment, as needed.

Ectocervical biopsies containing both epithelial and submucosal tissue were obtained from each participant by collecting two punches, each measuring 3 mm^2^, from the superior portion of the ectocervix using Schubert biopsy forceps (B. Braun Aesculap). One biopsy was immediately snap-frozen, cryopreserved, and stored at -80°C until used for *in situ* immunostaining. The other biopsy was immersed in RNAlater Stabilization Solution (QIAGEN, Hilden, Germany) and stored at -80°C until utilized for bulk RNA-seq analysis [31].

### RNA-seq Analysis

Barcoded pooled RNA-seq data of patients from the cohort described above are publicly available on the GEO (GSE217237). In this study, we utilized gene expression counts to compare HIV^+^FSWs and HIV^-^FSWs (**S6 Table**). DEGs were determined using a negative binomial generalized linear model from the edgeR package (4.2.0) [73], with significance defined as an FDR-adjusted P-value < 0.05. As participants were matched for age and time in sex work, these were not considered as confounders. Re-analysis of the RNA-sequencing data was performed excluding the two HIV^+^FSWs using DMPA. PCA was performed using the normalized count per million of the DEGs from all subjects.

To identify functional alterations associated with chronic HIV infection FEA and transcription factor prediction analysis were conducted on genes with an FDR-adjusted P-value < 0.1, following the strategy described by Tarca *et al*. [33], using the EnrichR API (accessed 21^st^ of Oct 2024). A background list was provided consisting of the 15435 genes detected in the current bulk RNA-sequencing dataset (**S7 Table**) [74–77]. Upregulated and downregulated genes underwent FEA separately against the GO Resource [34–36], KEGG [37–39], and MSigDB [40, 41] for the prediction of functional pathways. Transcription factors were predicted by analyzing DEGs against the TRRUST [43] and ARCHS4 [42] databases.

### *In situ* immunofluorescence staining

*In situ* immunofluorescence staining was conducted on 8-μm-thick cryopreserved ectocervical tissue sections to evaluate the expression and spatial distribution of the EJPs E-cadherin, claudin-1, DSG1, and ZO1, as well as the CD4 cell-surface marker. Dual staining for E-cadherin and CD4 was performed as previously described [78]. For triple staining of claudin-1, DSG1, and ZO1, tissue sections were fixed in 100% acetone and subsequently incubated with primary monoclonal antibodies, followed by addition of fluorophore-conjugated secondary antibodies (**S8 Table**). Following each step, sections were washed with phosphate-buffered saline containing 1% HEPES (HyClone, Nordic Biolabs, Täby, Sweden) and 0.1% saponin (Sigma-Aldrich, Solna, Sweden). Negative controls were incubated solely with fluorophore-conjugated secondary antibodies. All tissue sections were counterstained with 4’6-diamidino-2-phenylindole (DAPI; Invitrogen, Thermo Fischer Scientific, Waltham, MA, USA) and scanned as digital images using a Pannoramic 250 Flash Slide Scanner with a 20× objective (3DHISTECH Ltd, Budapest, Hungary) (**S8 Table**).

### Bioimage analysis of epithelial integrity

Two to six regions of interest (ROIs) were manually annotated per individual, depending on the size and quality of the biopsy in Caseviewer (version 2.4, 3DHISTECH Ltd). All ROIs were annotated blinded to avoid introducing bias. The epithelial compartment was outlined in FIJI (v.153c) [79] by delineating the superficial and basal borders of the epithelium (**Fig 4A and 4B**) and averaged 0.18 mm^2^ per ROI (**S8 Table).** The net-like structures of the junction protein strands were identified using a contrast-independent approach enhancing curve-linear structures (**Fig 4C–4E**) [80].

The epithelium was compartmentalized into three layers: superficial and basal layers devoid of staining and an intermediate (IM) layer represented by expression of the junction protein strands (**Fig 4F**). The identified protein strands of each EJP were classified as either fragmented or intact based on the size of the connected components (**Fig 4G**).

Using the identified intact net-like structures, the epithelium was divided into theoretical viral accessible versus protected regions using a flooding watershed transformation from the cervicovaginal lumen towards the basal membrane (**Fig 4H**). To further assess epithelial stability, the IM layer of each EJP was divided into fragmented and intact regions by watershed transformation to represent disrupted versus intact expression patterns of the protein strands (**Fig 4I**). Any breaks larger than 1 pixel (representing 0.325μm) were considered as a fragmentation of the protein strand and allowed for theoretical viral transmigration (**Fig 4L**). The percentage epithelial coverage of each layer was then calculated for the respective EJPs.

The total height of the epithelium was calculated by generating a Euclidian distance-transformed image and calculating the intensity (i.e., distance) from each point on the superficial border to each point on the basal border and vice versa. The height of the individual superficial, IM, and basal layers was thereafter measured in a similar fashion. The MFI of the IM layer and the whole epithelial compartment was calculated for each protein.

The identified protein strands of claudin-1, DSG1, and ZO1 were merged to perform a combined analysis for a more in-depth assessment of epithelial integrity (**Fig 4J–4K**). All measurements were conducted on the combined analysis, except for the MFI measurements due to technical restraints. For each sample, an average of the individual ROIs was calculated and used for statistical analysis and graphical presentation to better represent the whole epithelium (for further details see **S1 Appendix)**.

### Quantification and localization of CD4^+^ cells

Pixel-based segmentation was employed to evaluate the frequency of CD4^+^ cells within the epithelial compartment [78]. A white top-hat filter was applied for noise reduction, followed by image-dependent intensity thresholding. The area fraction occupied by CD4^+^ cells out of the epithelial area was used to quantify CD4^+^ cell frequency. Additionally, the distance of CD4^+^ cells to the superficial border was determined using a Euclidian distance transform to measure the intensity (i.e., distance).

### Statistical analysis

Categorical variables in the clinical data were analyzed using Pearson χ^2^ and Fisher’s exact tests. The significance of continuous variables in both clinical and imaging data was assessed using the Mann–Whitney U test, while correlations were determined using Spearman’s rank correlation coefficient. DEGs and FEA were considered significant at an FDR-adjusted *P-*value <0.05. In the case of correlations and imaging data, significance was set at a nominal *P-*value<0.05.

## Supporting information

S1 Fig Dimensionality reduction reveal no distinct separation between HIV^+^FSWs and HIV^-^FSWs. (PDF)

S2 Fig Plasma viral load did not correlate with *S100A7A*, *TFF2* or genes involved in epithelial structure. (PDF)

S3 Fig HIV^+^FSWs demonstrate an epithelial downregulation of DSG1. (PDF)

S4 Fig No difference in intra-epithelial CD4^+^ frequency or correlation to blood CD4^+^ cells observed between the HIV^+^- and HIV^-^FSWs. (PDF)

S1 Table Differentially expressed genes between HIV^+^FSWs and HIV^-^FSWs. (XLSX)

S2 Table Differentially expressed genes between HIV^+^FSWs and HIV^-^FSWs with DMPA users removed. (XLSX)

S3 Table GOslim enrichment of GO pathways. (XLSX)

S4 Table Functional enrichment analysis of genes with an FDR < 0.1 between HIV^+^FSWs and HIV^-^FSWs. (XLSX)

S5 Table Transcription factor prediction analysis of genes with an FDR < 0.1 between HIV^+^FSWs and HIV^-^FSWs. (XLSX)

S6 Table Study participants. (XLSX)

S7 Table Functional enrichment analysis background gene list. (XLSX)

S8 Table Antibody information, imaging settings and average epithelial ROI size. (XLSX)

S1 Appendix Bioimage analysis. (DOCX)

## References

[1] Kamat A, Misra V, Cassol E, Ancuta P, Yan Z, Li C, et al. A Plasma Biomarker Signature of Immune Activation in HIV Patients on Antiretroviral Therapy. PLoS One. 2012 Feb 17;7(2). Available from: /pmc/articles/PMC3281899/. doi: 10.1371/journal.pone.0030881 22363505 PMC3281899

[2] So-Armah KA, Tate JP, Chang CCH, Butt AA, Gerschenson M, Gibert CL, et al. Do Biomarkers of Inflammation, Monocyte Activation, and Altered Coagulation Explain Excess Mortality Between HIV Infected and Uninfected People? J Acquir Immune Defic Syndr. 2016 Jun 6;72(2):206. Available from: /pmc/articles/PMC4867134/. doi: 10.1097/QAI.0000000000000954 27824677 PMC4867134

[3] Brenchley JM, Price DA, Schacker TW, Asher TE, Silvestri G, Rao S, et al. Microbial translocation is a cause of systemic immune activation in chronic HIV infection. Nature Medicine 2006 12:12. 2006 Nov 19;12(12):1365–71. Available from: https://www.nature.com/articles/nm1511. doi: 10.1038/nm1511 17115046

[4] Giorgi J V., Hultin LE, McKeating JA, Johnson TD, Owens B, Jacobson LP, et al. Shorter Survival in Advanced Human Immunodeficiency Virus Type 1 Infection Is More Closely Associated with T Lymphocyte Activation than with Plasma Virus Burden or Virus Chemokine Coreceptor Usage. J Infect Dis. 1999 Apr 1;179(4):859–70. Available from: doi: 10.1086/314660 10068581

[5] Nasi M, De Biasi S, Gibellini L, Bianchini E, Pecorini S, Bacca V, et al. Ageing and inflammation in patients with HIV infection. Clin Exp Immunol. 2017 Jan 1;187(1):44. Available from: /pmc/articles/PMC5167025/. doi: 10.1111/cei.12814 27198731 PMC5167025

[6] Nasi M, Pinti M, Mussini C, Cossarizza A. Persistent inflammation in HIV infection: established concepts, new perspectives. Immunol Lett. 2014;161(2):184–8. Available from: https://pubmed.ncbi.nlm.nih.gov/24487059/. doi: 10.1016/j.imlet.2014.01.008 24487059

[7] Rajasuriar R, Khoury G, Kamarulzaman A, French MA, Cameron PU, Lewin SR. Persistent immune activation in chronic HIV infection: do any interventions work? AIDS. 2013 May 5;27(8):1199. Available from: /pmc/articles/PMC4285780/. doi: 10.1097/QAD.0b013e32835ecb8b 23324661 PMC4285780

[8] Hellerstein M, Hanley MB, Cesar D, Siler S, Papageorgopoulos C, Wieder E, et al. Directly measured kinetics of circulating T lymphocytes in normal and HIV-1-infected humans. Nature Medicine 1999 5:1. 1999;5(1):83–9. Available from: https://www.nature.com/articles/nm0199_83. doi: 10.1038/4772 9883844

[9] Del Cornò M, Cappon A, Donninelli G, Varano B, Marra F, Gessani S. HIV-1 gp120 signaling through TLR4 modulates innate immune activation in human macrophages and the biology of hepatic stellate cells. J Leukoc Biol. 2016 Sep 1;100(3):599–606. Available from: doi: 10.1189/jlb.4A1215-534R 26992429

[10] Sandler NG, Douek DC. Microbial translocation in HIV infection: causes, consequences and treatment opportunities. Nature Reviews Microbiology 2012 10:9. 2012 Aug 13;10(9):655–66. Available from: https://www.nature.com/articles/nrmicro2848. 10.1038/nrmicro2848 22886237

[11] Kobayashi A, Greenblatt RM, Anastos K, Minkoff H, Massad LS, Young M, et al. Functional Attributes of Mucosal Immunity in Cervical Intraepithelial Neoplasia and Effects of HIV Infection. Cancer Res. 2004 Sep 15;64(18):6766–74. Available from: /cancerres/article/64/18/6766/511676/Functional-Attributes-of-Mucosal-Immunity-in. doi: 10.1158/0008-5472.CAN-04-1091 15374995

[12] Gumbi PP, Nkwanyana NN, Bere A, Burgers WA, Gray CM, Williamson AL, et al. Impact of Mucosal Inflammation on Cervical Human Immunodeficiency Virus (HIV-1)-Specific CD8 T-Cell Responses in the Female Genital Tract during Chronic HIV Infection. J Virol. 2008 Sep;82(17):8529–36. Available from: https://journals.asm.org/doi/10.1128/jvi.00183-08. 18562528 10.1128/JVI.00183-08 PMC2519691

[13] Bere A, Tayib S, Kriek JM, Masson L, Jaumdally SZ, Barnabas SL, et al. Altered phenotype and function of NK cells infiltrating Human Papillomavirus (HPV)-associated genital warts during HIV infection. Clinical Immunology. 2014 Feb 1;150(2):210–9. doi: 10.1016/j.clim.2013.12.005 24440646

[14] Kriek JM, Jaumdally SZ, Masson L, Little F, Mbulawa Z, Gumbi PP, et al. Female genital tract inflammation, HIV co-infection and persistent mucosal Human Papillomavirus (HPV) infections. Virology. 2016 Jun 1;493:247–54. doi: 10.1016/j.virol.2016.03.022 27065342

[15] Gumbi PP, Jaumdally SZ, Salkinder AL, Burgers WA, Mkhize NN, Hanekom W, et al. CD4 T Cell Depletion at the Cervix during HIV Infection Is Associated with Accumulation of Terminally Differentiated T Cells. J Virol. 2011 Dec 15;85(24):13333. Available from: /pmc/articles/PMC3233181/. doi: 10.1128/JVI.05671-11 21994461 PMC3233181

[16] Hirbod T, Kimani J, Tjernlund A, Cheruiyot J, Petrova A, Ball TB, et al. Stable CD4 Expression and Local Immune Activation in the Ectocervical Mucosa of HIV-Infected Women. The Journal of Immunology. 2013 Oct 1;191(7):3948–54. Available from: doi: 10.4049/jimmunol.1301220 24006463

[17] Sawada N, Murata M, Kikuchi K, Osanai M, Tobioka H, Kojima T, et al. Tight junctions and human diseases. Medical Electron Microscopy. 2003 Sep;36(3):147–56. Available from: https://link.springer.com/article/10.1007/s00795-003-0219-y. 14505058 10.1007/s00795-003-0219-y

[18] Nazli A, Chan O, Dobson-Belaire WN, Ouellet M, Tremblay MJ, Gray-Owen SD, et al. Exposure to HIV-1 Directly Impairs Mucosal Epithelial Barrier Integrity Allowing Microbial Translocation. PLoS Pathog. 2010;6(4):e1000852. Available from: https://journals.plos.org/plospathogens/article?id=10.1371/journal.ppat.1000852. 20386714 10.1371/journal.ppat.1000852 PMC2851733

[19] Tugizov SM, Herrera R, Chin-Hong P, Veluppillai P, Greenspan D, Michael Berry J, et al. HIV-associated disruption of mucosal epithelium facilitates paracellular penetration by human papillomavirus. Virology. 2013 Nov;446(0):378–88. Available from: /pmc/articles/PMC3809142/. doi: 10.1016/j.virol.2013.08.018 24074602 PMC3809142

[20] Sufiawati I, Tugizov SM. HIV-induced matrix metalloproteinase-9 activation through mitogen-activated protein kinase signalling promotes HSV-1 cell-to-cell spread in oral epithelial cells. J Gen Virol. 2018;99(7):937. Available from: /pmc/articles/PMC6537617/. doi: 10.1099/jgv.0.001075 29775175 PMC6537617

[21] Nkwanyana NN, Gumbi PP, Roberts L, Denny L, Hanekom W, Soares A, et al. Impact of human immunodeficiency virus 1 infection and inflammation on the composition and yield of cervical mononuclear cells in the female genital tract. Immunology. 2009 Sep;128(1 Pt 2):e746. Available from: /pmc/articles/PMC2753906/. doi: 10.1111/j.1365-2567.2009.03077.x 19740336 PMC2753906

[22] Archary D, Liebenberg LJ, Werner L, Tulsi S, Majola N, Naicker N, et al. Randomized Cross-Sectional Study to Compare HIV-1 Specific Antibody and Cytokine Concentrations in Female Genital Secretions Obtained by Menstrual Cup and Cervicovaginal Lavage. PLoS One. 2015 Jul 6;10(7):e0131906. Available from: https://journals.plos.org/plosone/article?id=10.1371/journal.pone.0131906. 26147923 10.1371/journal.pone.0131906 PMC4492781

[23] UNAIDS data 2019 | UNAIDS. Available from: https://www.unaids.org/en/resources/documents/2019/2019-UNAIDS-data.

[24] Mbulawa ZZA, Marais DJ, Johnson LF, Coetzee D, Williamson AL. Impact of Human Immunodeficiency Virus on the Natural History of Human Papillomavirus Genital Infection in South African Men and Women. J Infect Dis. 2012 Jul 1;206(1):15–27. Available from: doi: 10.1093/infdis/jis299 22517913

[25] Nava-Memije K, Hernández-Cortez C, Ruiz-González V, Saldaña-Juárez CA, Medina-Islas Y, Dueñas-Domínguez RA, et al. Bacterial Vaginosis and Sexually Transmitted Infections in an HIV-Positive Cohort. Frontiers in Reproductive Health. 2021;3:660672. Available from: /pmc/articles/PMC9580688/. doi: 10.3389/frph.2021.660672 36303986 PMC9580688

[26] Castro JG, Alcaide ML. High Rates of Sexually Transmitted Infections in HIV-Infected Patients Attending an STI Clinic. South Med J. 2016 Jan 1;109(1):1. Available from: /pmc/articles/PMC4705853/. 26741862 10.14423/SMJ.0000000000000389 PMC4705853

[27] Lajoie J, Boily-Larouche G, Doering K, Cheruiyot J, Oyugi J, Broliden K, et al. Improving Adherence to Post-Cervical Biopsy Sexual Abstinence in Kenyan Female Sex Workers. American Journal of Reproductive Immunology. 2016 Jul 1;76(1):82. Available from: /pmc/articles/PMC5089664/. doi: 10.1111/aji.12520 27221472 PMC5089664

[28] Edfeldt G, Kaldhusdal V, Czarnewski P, Bradley F, Bergström S, Lajoie J, et al. Distinct cervical tissue-adherent and luminal microbiome communities correlate with mucosal host gene expression and protein levels in Kenyan sex workers. Microbiome. 2023 Dec 1;11(1):1–22. Available from: https://microbiomejournal.biomedcentral.com/articles/10.1186/s40168-023-01502-4. 37004130 10.1186/s40168-023-01502-4 PMC10064689

[29] Hoffmann W. TFF2, a MUC6-binding lectin stabilizing the gastric mucus barrier and more (Review). Int J Oncol. 2015 Sep 1;47(3):806–16. Available from: http://www.spandidos-publications.com/10.3892/ijo.2015.3090/abstract. 26201258 10.3892/ijo.2015.3090

[30] Chu S, Moujaber O, Lemay S, Stochaj U. Multiple pathways promote microtubule stabilization in senescent intestinal epithelial cells. npj Aging 2022 8:1. 2022 Dec 16;8(1):1–12. Available from: https://www.nature.com/articles/s41514-022-00097-8. doi: 10.1038/s41514-022-00097-8 36526654 PMC9758230

[31] Bradley F, Boger MF, Kaldhusdal V, Åhlberg A, Edfeldt G, Lajoie J, et al. Multi-omics analysis of the cervical epithelial integrity of women using depot medroxyprogesterone acetate. PLoS Pathog. 2022 May 1;18(5). Available from: /pmc/articles/PMC9119532/. doi: 10.1371/journal.ppat.1010494 35533147 PMC9119532

[32] Zalenskaya IA, Chandra N, Yousefieh N, Fang X, Adedipe OE, Jackson SS, et al. Use of contraceptive depot medroxyprogesterone acetate is associated with impaired cervicovaginal mucosal integrity. J Clin Invest. 2018 Sep 9;128(10):4622. Available from: /pmc/articles/PMC6159996/. doi: 10.1172/JCI120583 30222141 PMC6159996

[33] Tarca AL, Bhatti G, Romero R. A Comparison of Gene Set Analysis Methods in Terms of Sensitivity, Prioritization and Specificity. PLoS One. 2013 Nov 15;8(11):79217. Available from: /pmc/articles/PMC3829842/. doi: 10.1371/journal.pone.0079217 24260172 PMC3829842

[34] Thomas PD, Ebert D, Muruganujan A, Mushayahama T, Albou LP, Mi H. PANTHER: Making genome-scale phylogenetics accessible to all. Protein Science. 2022 Jan 1;31(1):8–22. Available from: https://onlinelibrary.wiley.com/doi/full/10.1002/pro.4218. 34717010 10.1002/pro.4218 PMC8740835

[35] Ashburner M, Ball CA, Blake JA, Botstein D, Butler H, Cherry JM, et al. Gene Ontology: tool for the unification of biology. Nature Genetics 2000 25:1. 2000 May;25(1):25–9. Available from: https://www.nature.com/articles/ng0500_25. 10.1038/75556 PMC3037419 10802651

[36] Consortium TGO, Aleksander SA, Balhoff J, Carbon S, Cherry JM, Drabkin HJ, et al. The Gene Ontology knowledgebase in 2023. Genetics. 2023 May 4;224(1). Available from: 10.1093/genetics/iyad031. 36866529 PMC10158837

[37] Kanehisa M, Goto S. KEGG: Kyoto Encyclopedia of Genes and Genomes. Nucleic Acids Res. 2000 Jan 1;28(1):27. Available from: /pmc/articles/PMC102409/. doi: 10.1093/nar/28.1.27 10592173 PMC102409

[38] Kanehisa M. Toward understanding the origin and evolution of cellular organisms. Protein Sci. 2019 Nov 1;28(11):1947. Available from: /pmc/articles/PMC6798127/. doi: 10.1002/pro.3715 31441146 PMC6798127

[39] Kanehisa M, Furumichi M, Sato Y, Kawashima M, Ishiguro-Watanabe M. KEGG for taxonomy-based analysis of pathways and genomes. Nucleic Acids Res. 2023 Jan 1;51(D1):D587. Available from: /pmc/articles/PMC9825424/. doi: 10.1093/nar/gkac963 36300620 PMC9825424

[40] Liberzon A, Birger C, Thorvaldsdóttir H, Ghandi M, Mesirov JP, Tamayo P. The Molecular Signatures Database (MSigDB) hallmark gene set collection. Cell Syst. 2015 Dec 12;1(6):417. Available from: /pmc/articles/PMC4707969/. doi: 10.1016/j.cels.2015.12.004 26771021 PMC4707969

[41] Subramanian A, Tamayo P, Mootha VK, Mukherjee S, Ebert BL, Gillette MA, et al. Gene set enrichment analysis: A knowledge-based approach for interpreting genome-wide expression profiles. Proc Natl Acad Sci U S A. 2005 Oct 25;102(43):15545–50. Available from: https://www.pnas.org/doi/abs/10.1073/pnas.0506580102. 16199517 10.1073/pnas.0506580102 PMC1239896

[42] Lachmann A, Torre D, Keenan AB, Jagodnik KM, Lee HJ, Wang L, et al. Massive mining of publicly available RNA-seq data from human and mouse. Nature Communications 2018 9:1. 2018 Apr 10;9(1):1–10. Available from: https://www.nature.com/articles/s41467-018-03751-6. doi: 10.1038/s41467-018-03751-6 29636450 PMC5893633

[43] Han H, Cho JW, Lee S, Yun A, Kim H, Bae D, et al. TRRUST v2: an expanded reference database of human and mouse transcriptional regulatory interactions. Nucleic Acids Res. 2018 Jan 4;46(D1):D380–6. Available from: doi: 10.1093/nar/gkx1013 29087512 PMC5753191

[44] Winandy S, Wu P, Georgopoulos K. A Dominant Mutation in the lkaros Gene Leads to Rapid Development of Leukemia and Lymphoma. Cell. 1995;83:289–99. 7585946 10.1016/0092-8674(95)90170-1

[45] Wei Y, Davenport TC, Collora JA, Ma HK, Pinto-Santini D, Lama J, et al. Single-cell epigenetic, transcriptional, and protein profiling of latent and active HIV-1 reservoir revealed that IKZF3 promotes HIV-1 persistence. Immunity. 2023 Nov 14;56(11):2584–2601.e7. doi: 10.1016/j.immuni.2023.10.002 37922905 PMC10843106

[46] Ne E, Crespo R, Izquierdo-Lara R, Rao S, Koçer S, Górska A, et al. Catchet-MS identifies IKZF1-targeting thalidomide analogues as novel HIV-1 latency reversal agents. Nucleic Acids Res. 2022 Jun 6;50(10):5577. Available from: /pmc/articles/PMC9177988/. doi: 10.1093/nar/gkac407 35640596 PMC9177988

[47] Wilanowski T, Caddy J, Ting SB, Hislop NR, Cerruti L, Auden A, et al. Perturbed desmosomal cadherin expression in grainy head-like 1-null mice. EMBO J. 2008 Mar 3;27(6):886. Available from: /pmc/articles/PMC2274933/. doi: 10.1038/emboj.2008.24 18288204 PMC2274933

[48] Sen GL, Boxer LD, Webster DE, Bussat RT, Qu K, Zarnegar BJ, et al. ZNF750 Is a p63 Target Gene that Induces KLF4 to Drive Terminal Epidermal Differentiation. Dev Cell. 2012 Mar 13;22(3):669–77. doi: 10.1016/j.devcel.2011.12.001 22364861 PMC3306457

[49] Wei X, Lai Y, Li B, Qin L, Xu Y, Lin S, et al. CRISPR/Cas9-Mediated Deletion of Foxn1 in NOD/SCID/IL2rg−/− Mice Results in Severe Immunodeficiency. Scientific Reports 2017 7:1. 2017 Aug 10;7(1):1–12. Available from: https://www.nature.com/articles/s41598-017-08337-8. doi: 10.1038/s41598-017-08337-8 28798321 PMC5552779

[50] Buggert M, Tauriainen J, Yamamoto T, Frederiksen J, Ivarsson MA, Michaëlsson J, et al. T-bet and Eomes Are Differentially Linked to the Exhausted Phenotype of CD8+ T Cells in HIV Infection. PLoS Pathog. 2014;10(7). Available from: /pmc/articles/PMC4102564/. 10.1371/journal.ppat.1004251 PMC4102564 25032686

[51] Roberts JN, Buck CB, Thompson CD, Kines R, Bernardo M, Choyke PL, et al. Genital transmission of HPV in a mouse model is potentiated by nonoxynol-9 and inhibited by carrageenan. Nature Medicine 2007 13:7. 2007 Jul 1;13(7):857–61. Available from: https://www.nature.com/articles/nm1598. 10.1038/nm1598 17603495

[52] Carias AM, Mccoombe S, Mcraven M, Anderson M, Galloway N, Vandergrift N, et al. Defining the Interaction of HIV-1 with the Mucosal Barriers of the Female Reproductive Tract. 2013; Available from: http://dx.doi.org/10.1128. 10.1128/JVI.01377-13 PMC3807311 23966398

[53] Gibbs A, Hirbod T, Li Q, Bohman K, Ball TB, Plummer FA, et al. Presence of CD8+ T cells in the ectocervical mucosa correlates with genital viral shedding in HIV-infected women despite a low prevalence of HIV RNA-expressing cells in the tissue. J Immunol. 2014 Apr 15;192(8):3947–57. Available from: https://pubmed.ncbi.nlm.nih.gov/24639358/. doi: 10.4049/jimmunol.1302826 24639358 PMC4098133

[54] Sweet K, Bosire C, Sanusi B, Sherrod CJ, Kwatampora J, Waweru W, et al. Prevalence, incidence, and distribution of human papillomavirus types in female sex workers in Kenya. Int J STD AIDS. 2020 Feb 1;31(2):109–18. Available from: https://journals.sagepub.com/doi/10.1177/0956462419884454. 31948341 10.1177/0956462419884454 PMC7031817

[55] Rio MC, Chenard MP, Wolf C, Marcellin L, Tomasetto C, Lathe R, et al. Induction of pS2 and hSP genes as markers of mucosal ulceration of the digestive tract. Gastroenterology. 1991 Feb 1;100(2):375–9. doi: 10.1016/0016-5085(91)90205-y 1985035

[56] Viby NE, Pedersen L, Lund TK, Kissow H, Backer V, Nexø E, et al. Trefoil factor peptides in serum and sputum from subjects with asthma and COPD. Clin Respir J. 2015 Jul 1;9(3):322–9. Available from: https://onlinelibrary.wiley.com/doi/full/10.1111/crj.12146. 24720774 10.1111/crj.12146

[57] Buzzelli JN, Chalinor H V., Pavlic DI, Sutton P, Menheniott TR, Giraud AS, et al. IL33 Is a Stomach Alarmin That Initiates a Skewed Th2 Response to Injury and Infection. Cell Mol Gastroenterol Hepatol. 2015 Mar 1;1(2):203. Available from: /pmc/articles/PMC5301136/. doi: 10.1016/j.jcmgh.2014.12.003 28210674 PMC5301136

[58] Nazli A, Kafka JK, Ferreira VH, Anipindi V, Mueller K, Osborne BJ, et al. HIV-1 gp120 Induces TLR2- and TLR4-Mediated Innate Immune Activation in Human Female Genital Epithelium. The Journal of Immunology. 2013 Oct 15;191(8):4246–58. Available from: 10.4049/jimmunol.1301482. 24043886

[59] McNab F, Mayer-Barber K, Sher A, Wack A, O’Garra A. Type I interferons in infectious disease. Nature Reviews Immunology 2015 15:2. 2015 Jan 23;15(2):87–103. Available from: https://www.nature.com/articles/nri3787. doi: 10.1038/nri3787 25614319 PMC7162685

[60] Duffy P, Wang X, Lin PH, Yao Q, Chen C. HIV Nef Protein Causes Endothelial Dysfunction in Porcine Pulmonary Arteries and Human Pulmonary Artery Endothelial Cells. J Surg Res. 2009 Oct;156(2):257. Available from: /pmc/articles/PMC2760402/. doi: 10.1016/j.jss.2009.02.005 19540523 PMC2760402

[61] Gibbs A, Buggert M, Edfeldt G, Ranefall P, Introini A, Cheuk S, et al. Human Immunodeficiency Virus-Infected Women Have High Numbers of CD103−CD8+ T Cells Residing Close to the Basal Membrane of the Ectocervical Epithelium. J Infect Dis. 2018 Jul 2;218(3):453–65. Available from: https://academic.oup.com/jid/article/218/3/453/4767924. doi: 10.1093/infdis/jix661 29272532

[62] Jabri B, Selby JM, Negulescu H, Lee L, Roberts AI, Beavis A, et al. TCR specificity dictates CD94/NKG2A expression by human CTL. Immunity. 2002 Oct 1;17(4):487–99. Available from: http://www.cell.com/article/S1074761302004272/fulltext. doi: 10.1016/s1074-7613(02)00427-2 12387742

[63] Roff SR, Noon-Song EN, Yamamoto JK. The Significance of Interferon-γ in HIV-1 Pathogenesis, Therapy, and Prophylaxis. Front Immunol. 2013;4(DEC). Available from: /pmc/articles/PMC3888948/. 10.3389/fimmu.2013.00498 PMC3888948 24454311

[64] Tugizov S. Human immunodeficiency virus-associated disruption of mucosal barriers and its role in HIV transmission and pathogenesis of HIV/AIDS disease. Tissue Barriers. 2016 Jul 2;4(3). Available from: /pmc/articles/PMC4993574/. doi: 10.1080/21688370.2016.1159276 27583187 PMC4993574

[65] Deng Z, Cangkrama M, Butt T, Jane SM, Carpinelli MR. Grainyhead-like transcription factors: guardians of the skin barrier. Vet Dermatol. 2021 Dec 1;32(6):553–e152. Available from: https://onlinelibrary.wiley.com/doi/full/10.1111/vde.12956. 33843098 10.1111/vde.12956

[66] Sen GL, Boxer LD, Webster DE, Bussat RT, Qu K, Zarnegar BJ, et al. ZNF750 is a p63 Target Gene that Induces KLF4 to Drive Terminal Epidermal Differentiation. Dev Cell. 2012 Mar 3;22(3):669. Available from: /pmc/articles/PMC3306457/. doi: 10.1016/j.devcel.2011.12.001 22364861 PMC3306457

[67] Lien K, Mayer W, Herrera R, Rosbe K, Tugizov SM. HIV-1 proteins gp120 and tat induce the epithelial–mesenchymal transition in oral and genital mucosal epithelial cells. PLoS One. 2019 Dec 1;14(12). Available from: /pmc/articles/PMC6927651/. doi: 10.1371/journal.pone.0226343 31869348 PMC6927651

[68] Sufiawati I, Tugizov SM. HIV-associated disruption of tight and adherens junctions of oral epithelial cells facilitates HSV-1 infection and spread. PLoS One. 2014 Feb 21;9(2). Available from: https://pubmed.ncbi.nlm.nih.gov/24586397/. doi: 10.1371/journal.pone.0088803 24586397 PMC3931628

[69] Mehandru S, Poles MA, Tenner-Racz K, Horowitz A, Hurley A, Hogan C, et al. Primary HIV-1 Infection Is Associated with Preferential Depletion of CD4+ T Lymphocytes from Effector Sites in the Gastrointestinal Tract. J Exp Med. 2004 Sep 9;200(6):761. Available from: /pmc/articles/PMC2211967/. doi: 10.1084/jem.20041196 15365095 PMC2211967

[70] Gibbs A, Leeansyah E, Introini A, Paquin-Proulx D, Hasselrot K, Andersson E, et al. MAIT cells reside in the female genital mucosa and are biased towards IL-17 and IL-22 production in response to bacterial stimulation. Mucosal Immunol. 2017 Jan 1;10(1):35–45. Available from: /pmc/articles/PMC5053908/. doi: 10.1038/mi.2016.30 27049062 PMC5053908

[71] Hirbod T, Kaldensjö T, Lopalco L, Klareskog E, Andersson S, Uberti-Foppa C, et al. Abundant and superficial expression of C-type lectin receptors in ectocervix of women at risk of HIV infection. J Acquir Immune Defic Syndr. 2009 Jul [cited 2024 Apr 21];51(3):239–47. Available from: https://pubmed.ncbi.nlm.nih.gov/19363450/. doi: 10.1097/QAI.0b013e3181a74f89 19363450

[72] Cantero-Pérez J, Grau-Expósito J, Serra-Peinado C, Rosero DA, Luque-Ballesteros L, Astorga-Gamaza A, et al. Resident memory T cells are a cellular reservoir for HIV in the cervical mucosa. Nat Commun. 2019 Dec 1;10(1):1–16. Available from: 10.1038/s41467-019-12732-2 31628331 PMC6802119

[73] Robinson MD, McCarthy DJ, Smyth GK. edgeR: a Bioconductor package for differential expression analysis of digital gene expression data. Bioinformatics. 2010 Jan 1;26(1):139. Available from: /pmc/articles/PMC2796818/. doi: 10.1093/bioinformatics/btp616 19910308 PMC2796818

[74] Xie Z, Bailey A, Kuleshov M V., Clarke DJB, Evangelista JE, Jenkins SL, et al. Gene Set Knowledge Discovery with Enrichr. Curr Protoc. 2021 Mar 1;1(3):e90. Available from: /pmc/articles/PMC8152575/. doi: 10.1002/cpz1.90 33780170 PMC8152575

[75] An R interface to the Enrichr database. [cited 2024 Sep 27]. Available from: https://cran.r-project.org/web/packages/enrichR/vignettes/enrichR.html.

[76] Chen EY, Tan CM, Kou Y, Duan Q, Wang Z, Meirelles G V., et al. Enrichr: interactive and collaborative HTML5 gene list enrichment analysis tool. BMC Bioinformatics. 2013 Apr 15;14:128. Available from: /pmc/articles/PMC3637064/. doi: 10.1186/1471-2105-14-128 23586463 PMC3637064

[77] Kuleshov M V., Jones MR, Rouillard AD, Fernandez NF, Duan Q, Wang Z, et al. Enrichr: a comprehensive gene set enrichment analysis web server 2016 update. Nucleic Acids Res. 2016 Jul 7;44:W90. Available from: /pmc/articles/PMC4987924/. doi: 10.1093/nar/gkw377 27141961 PMC4987924

[78] Edfeldt G, Lajoie J, Röhl M, Oyugi J, Åhlberg A, Khalilzadeh-Binicy B, et al. Regular Use of Depot Medroxyprogesterone Acetate Causes Thinning of the Superficial Lining and Apical Distribution of Human Immunodeficiency Virus Target Cells in the Human Ectocervix. J Infect Dis. 2022 Apr 4;225(7):1151. Available from: /pmc/articles/PMC8974825/. doi: 10.1093/infdis/jiaa514 32780807 PMC8974825

[79] Schindelin J, Arganda-Carreras I, Frise E, Kaynig V, Longair M, Pietzsch T, et al. Fiji: an open-source platform for biological-image analysis. Nature Methods 2012 9:7. 2012 Jun 28;9(7):676–82. Available from: https://www.nature.com/articles/nmeth.2019. doi: 10.1038/nmeth.2019 22743772 PMC3855844

[80] Obara B, Fricker M, Gavaghan D, Grau V. Contrast-independent curvilinear structure detection in biomedical images. IEEE Transactions on Image Processing. 2012 May;21(5):2572–81. doi: 10.1109/TIP.2012.2185938 22287240

