# Supplementary material for: Sustained immune activation and impaired epithelial barrier integrity in the ectocervix of women with chronic HIV infection
Source: PLoS Pathog. 2024 Nov 19;20(11):e1012709. doi: 10.1371/journal.ppat.1012709 (PMC11614238; doi:10.1371/journal.ppat.1012709)
Supplement: S1 Fig — (PDF) [file ppat.1012709.s001.pdf]

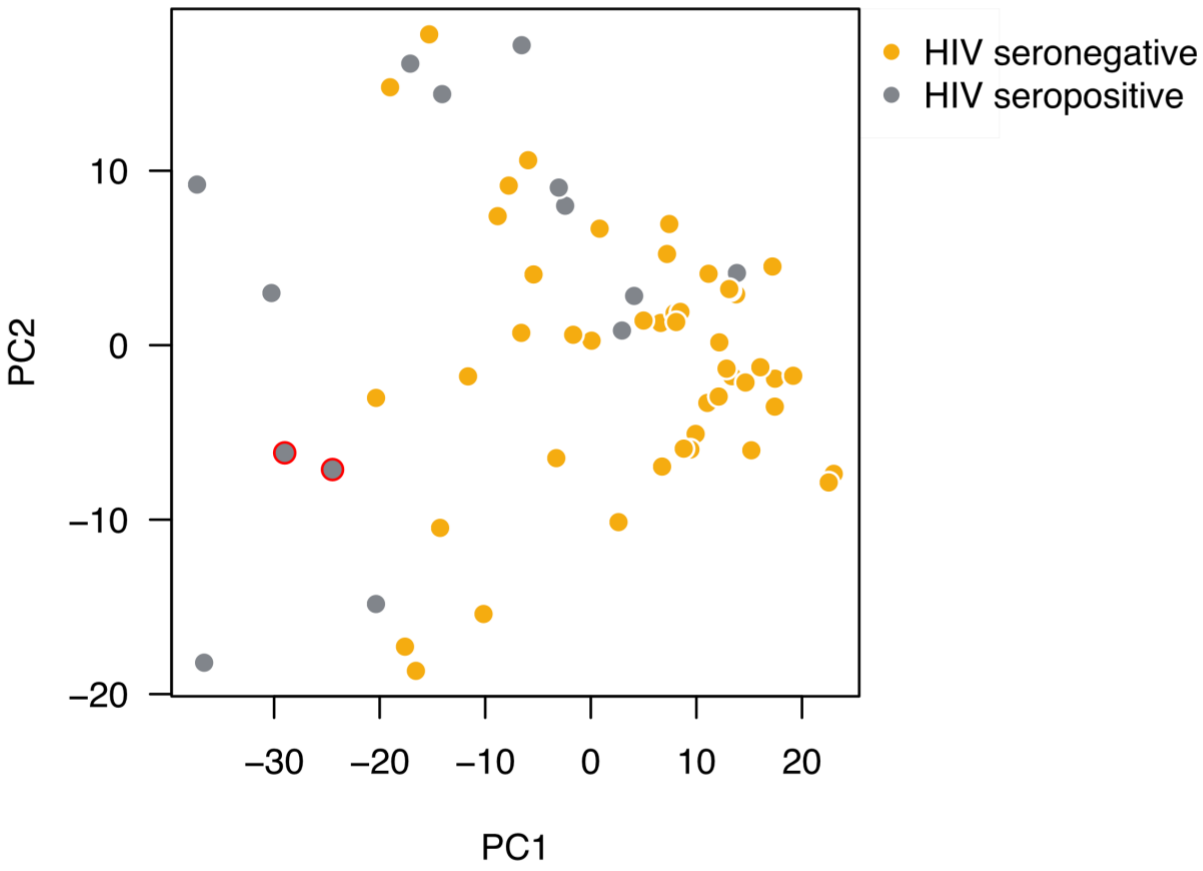

Supplementary Figure 1. Dimensionality reduction reveal no distinct separation between HIV⁺FSWs and HIV⁻FSWs.

Unsupervised PCA on the differentially expressed gene counts. Samples are colour coded by study group, HIV$^+$FSWs (n=14, grey) and HIV$^-$FSWs (n=47, orange). HIV$^+$FSWs using DMPA are encircled in red. FSW: Female sex worker. PCA: Principal component analysis. DMPA: Depot medroxyprogesterone acetate.
