# Supplementary material for: Sustained immune activation and impaired epithelial barrier integrity in the ectocervix of women with chronic HIV infection
Source: PLoS Pathog. 2024 Nov 19;20(11):e1012709. doi: 10.1371/journal.ppat.1012709 (PMC11614238; doi:10.1371/journal.ppat.1012709)
Supplement: S2 Fig — (PDF) [file ppat.1012709.s002.pdf]

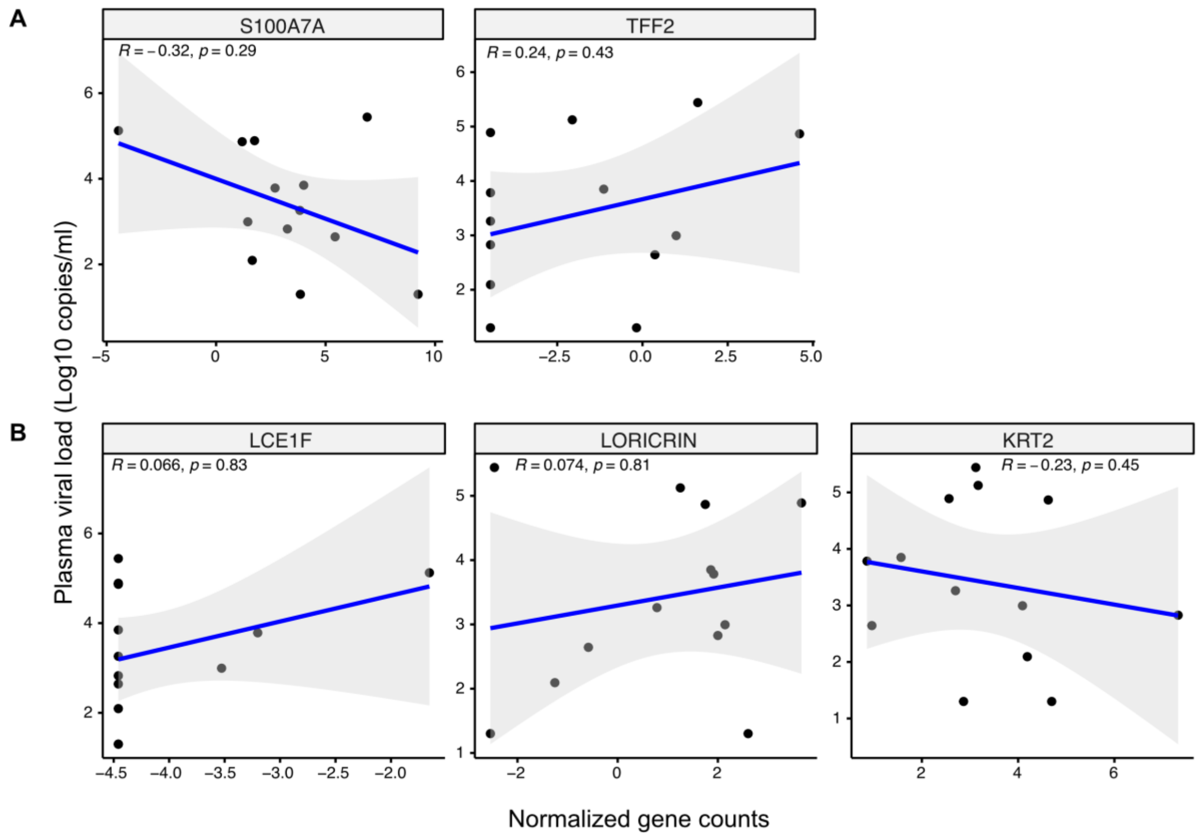

Supplementary Figure 2. Plasma viral load did not correlate with *S100A7A*, *TFF2* or genes involved in epithelial structure.

Correlation analysis of plasma viral load and the top upregulated DEGs ***A***, *S100A7A* and *TFF2*, and ***B***, the downregulated DEGs *LCE1F*, *LORICRIN* and *KRT2* involved in epithelial structure selected based on FC within HIV^+FSWs. The graphs illustrate Spearman correlation between gene count and plasma viral load (counts/ml) with a linear regression line, the 95% confidence interval and all individual datapoints. The log_{10} plasma viral load is shown on the y axis and the normalized gene count is shown on the x axis. P<0.05 was considered significant.
DEG; differentially expressed gene. FC; Fold-change.
