# Supplementary material for: Sustained immune activation and impaired epithelial barrier integrity in the ectocervix of women with chronic HIV infection
Source: PLoS Pathog. 2024 Nov 19;20(11):e1012709. doi: 10.1371/journal.ppat.1012709 (PMC11614238; doi:10.1371/journal.ppat.1012709)
Supplement: S3 Fig — (PDF) [file ppat.1012709.s003.pdf]

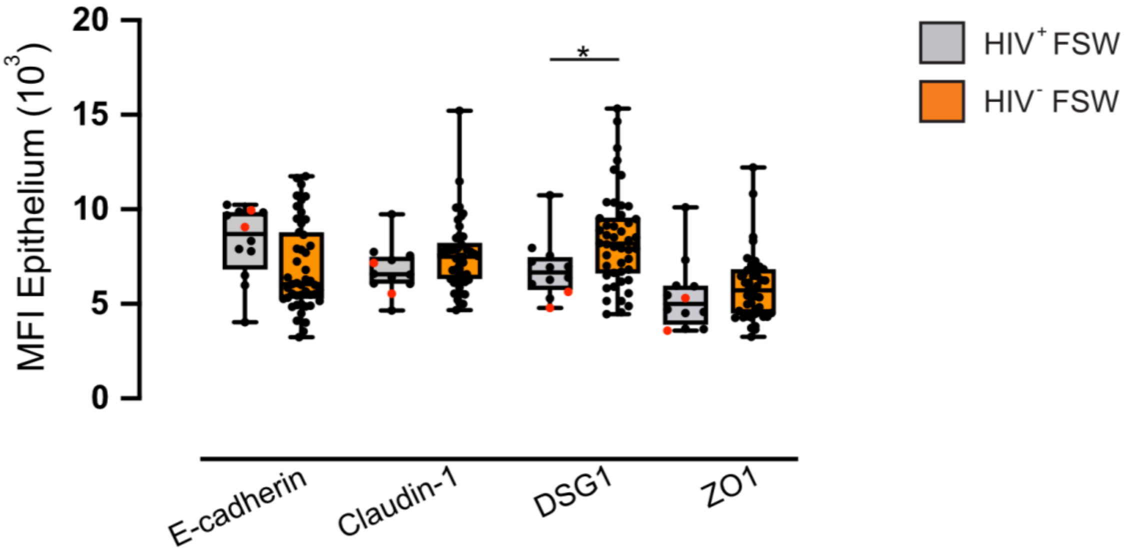

Supplementary Figure 3. HIV^{+}FSWs demonstrate an epithelial downregulation of DSG1

Boxplots demonstrating the MFI of each EJP within the whole epithelial compartment. Samples from HIV^{+}FSWs (n=12) are shown in grey and HIV^{-}FSWs (n=46) are shown in orange. Statistical analysis was performed using Mann Whitney U comparison. Boxplots demonstrate median and IQR while whiskers show the full range. HIV^{+}FSWs using DMPA are highlighted in red. $P<0.05$ was considered statistically significant. DSG1: Desmoglein-1. ZO1: Zonula occludens-1. MFI: Mean fluorescent intensity. EJP: Epithelial junction protein. FSW: Female sex worker. IQR: Interquartile range. DMPA: Depot medroxyprogesterone acetate. *: $P<0.05$.
