# Supplementary material for: Sustained immune activation and impaired epithelial barrier integrity in the ectocervix of women with chronic HIV infection
Source: PLoS Pathog. 2024 Nov 19;20(11):e1012709. doi: 10.1371/journal.ppat.1012709 (PMC11614238; doi:10.1371/journal.ppat.1012709)
Supplement: S4 Fig — (PDF) [file ppat.1012709.s004.pdf]

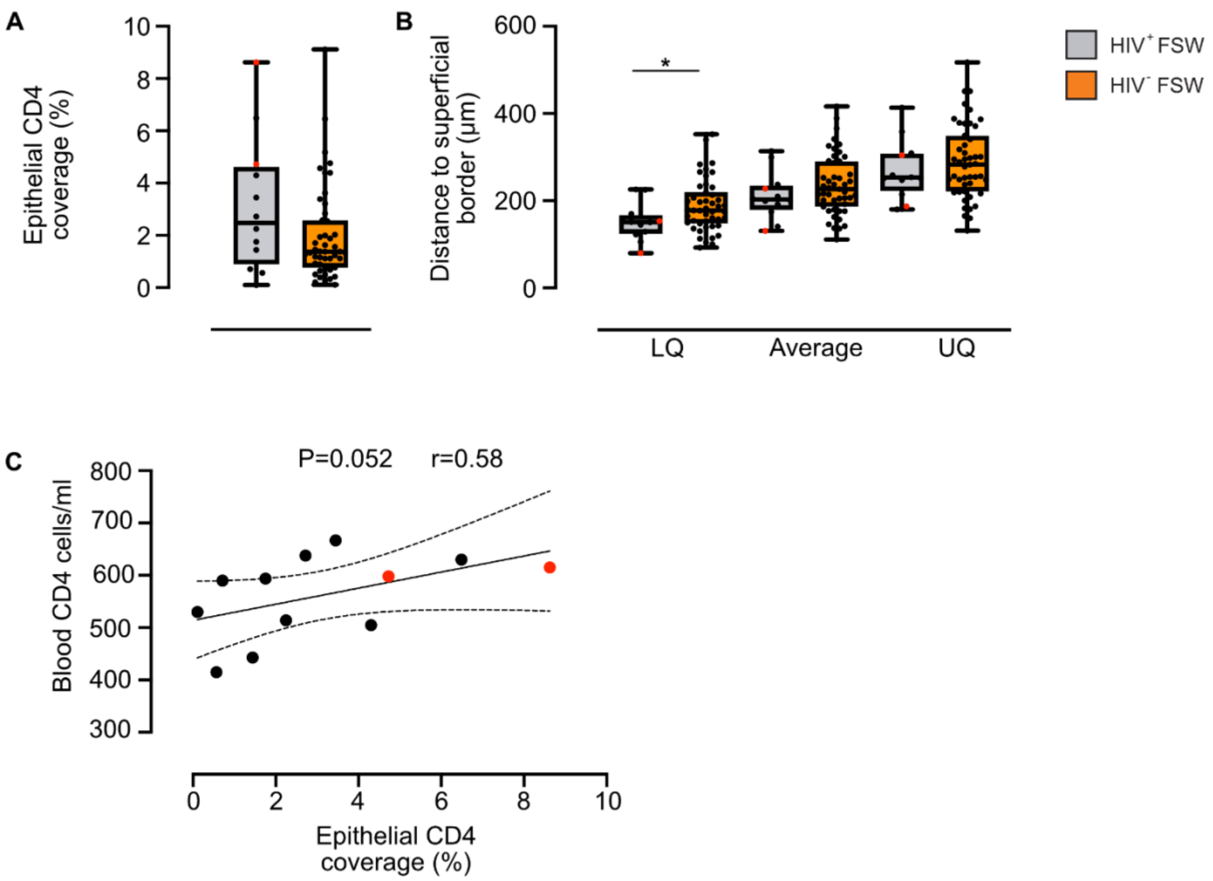

Supplementary Figure 4. No difference in intra-epithelial CD4$^+$ frequency or correlation to blood CD4$^+$ cells observed between the HIV$^+$- and HIV$^-$FSWs.

Assessement of the ectocervical CD4$^+$ cell population within HIV$^+$FSW (n=12, grey) and HIV$^-$ FSWs (n=47, orange). ***A***, Boxplots demonstrating the epithelial coverage of CD4$^+$ cells. ***B***, Boxplots demonstrating the LQ, average and UQ distance of the CD4$^+$ cells to the superficial border of the ectocervical epithelium. ***C***, Correlation between blood CD4$^+$ cells and CD4$^+$ cell coverage of the epithelium within the HIV$^+$FSW. Statistical analysis was performed using Mann Whitney U comparison and spearman correlation with a linear regression line and 95% confidence interval. Boxplots demonstrate median and IQR while whiskers show the full range. HIV$^+$FSWs using DMPA are highlighted in red. P<0.05 was considered statistically significant. LQ: Lower quartile. UQ: Upper quartile. FSW: Female sex worker. DMPA: Depot medroxyprogesterone acetate. *: P<0.05.
