# Supplementary material for: Sustained immune activation and impaired epithelial barrier integrity in the ectocervix of women with chronic HIV infection
Source: PLoS Pathog. 2024 Nov 19;20(11):e1012709. doi: 10.1371/journal.ppat.1012709 (PMC11614238; doi:10.1371/journal.ppat.1012709)
Supplement: S1 Appendix — (DOCX) [file ppat.1012709.s013.docx]

**Bioimage analysis**

The epithelial compartment was manually outlined in Caseviewer (version 2.4, 3DHistech Ltd, Budapest, Hungary). Between two to six regions of interest (ROIs) were generated per tissue section. Tissue regions containing damaged epithelium, artefacts, or cysts were excluded from analysis. Images of each ROI were exported as individual .tif grey-scale images, one for each channel (DAPI, FITC, Cy3 and Cy5) and analysed independently. All ROIs were selected blinded as to not introduce any bias. Image analysis was performed using MatLAB (vR2020b, MathWorks, Natick, MA, US) and Fiji (v.153c).

- **Epithelial layer compartmentalization**

The apical border and the basal membrane were manually annotated to enable analysis of the epithelial compartment (i.e., excluding the cervical submucosa and lumen from analysis) (**Fig 1A-B**). A pre-processing step was next used to enhance curvilinear structures [1] to highlight the net-like shape of the junction proteins (**Fig 1C**). This approach is a contrast-independent method based on the concept of local phase congruency, which offers a better performance than other traditional intensity-based methods to detect curvilinear objects [1].

The enhanced image was then segmented using the Multi Otsu Threshold plugin for Fiji [2] with three classes, which were further grouped in foreground, corresponding to the junction proteins, and background **(S1 Table)**. On the resulting binary image, a correction factor was applied to the thresholding result in order to fine tune the segmentation. Following this step, the Euclidean distance transform was applied to the binary mask and a distance threshold was used to dilate the region corresponding to the junction proteins. Since the net-like structure can be very fragmented, this dilation allowed to obtain bigger connected components encompassing all the positive regions of the net-like image. Finally, these components were filtered by size and the final mask of the junction proteins was defined **(Fig 1D-E)**.

Following the previous work of *Edfeldt et. al* [3]*,* the epithelium was compartmentalized into three separate layers. The binary mask obtained from the segmentation of the junction proteins was defined as the intermediate layer, which contains the in situ-stained junction proteins (E-cadherin, claudin-1, DSG1 or ZO1). The superficial layer (towards the lumen) and the basal layer (towards the submucosa) were devoid of in situ stained junctional proteins **(Fig 1F).** If the binary mask contains more than one connected, a minimum size criterion is applied in order to select which components will be part of the intermediate layer. The height of the epithelium as well as the height of the three individual epithelial layers were calculated using the Euclidean distance transform. This technique generates a distance mapping image. Explicitly, the intensity (i.e., distance) from the apical border to the basal border and vice versa was determined.

- **Epithelial integrity analysis**

For a more detailed assessment of the spatial localization and epithelial integrity of each junctional protein, the area and relative area (%) occupied by each layer were calculated. The segmented intermediate layer was used to assess the integrity of the epithelium. The segmented net within the intermediate layer was classed as either intact or fragmented based on size exclusion of the connected components (**Fig 1G**).

To generate the intact net, a morphological reconstruction [4,5] was applied using the apical epithelial boundary as marker and the intermediate layer as mask. This process resulted in the reconstruction of the region between the apical line and the intact region. To assess the epithelial accessibility for an incoming viral particle a flooding watershed transformation was performed on the EJP layer from the apical boarder towards the basal membrane based on the intact region of the EJP to theoretically measure the stability of the epithelium (**Figure 1H**).

An additional flooding watershed transformation was then applied to the binary mask of the net-like shape of the junction proteins, where all holes present within the intact net were filled, followed by removal of spurs. The fragmented layer was thereafter generated by subtracting the intact layer from the intermediate layer **(Figure I)**

The mean fluorescent intensity (MFI) was measured in the whole, non-compartmentalized epithelial region and within the compartmentalized intermediate layer for each of the four junctional proteins assessed.

- **Combined epithelial junction protein analysis**

The identified net-like structure of claudin-1, DSG1 and ZO1 were merged to perform a combined analysis of the three junctional proteins simultaneously **(Figure 1 J-K)** creating an epithelial junctional protein (EJP) layer. All aforementioned measurements were thereafter performed on the EJP layer, except for the MFI measurements.

**References**

1. Obara B, Fricker M, Gavaghan D, Grau V. Contrast-independent curvilinear structure detection in biomedical images. IEEE Transactions on Image Processing. **2012**; 21(5):2572–2581.

2. Liao P-S, Chen T-S, Chung P. A Fast Algorithm for Multilevel Thresholding. J Inf Sci Eng. **2001**; .

3. Edfeldt G, Lajoie J, Röhl M, et al. Regular Use of Depot Medroxyprogesterone Acetate Causes Thinning of the Superficial Lining and Apical Distribution of Human Immunodeficiency Virus Target Cells in the Human Ectocervix. J Infect Dis [Internet]. Oxford University Press; **2022** [cited 2023 Aug 16]; 225(7):1151. Available from: /pmc/articles/PMC8974825/

4. Breen EJ, Jones R. Attribute Openings, Thinnings, and Granulometries. Computer Vision and Image Understanding. Academic Press; **1996**; 64(3):377–389.

5. Legland D, Arganda-Carreras I, Andrey P. MorphoLibJ: integrated library and plugins for mathematical morphology with ImageJ. Bioinformatics [Internet]. Oxford Academic; **2016** [cited 2023 Sep 5]; 32(22):3532–3534. Available from: https://dx.doi.org/10.1093/bioinformatics/btw413
